# Telomere attrition rates are associated with weather conditions and predict productive lifespan in dairy cattle

**DOI:** 10.1038/s41598-021-84984-2

**Published:** 2021-03-10

**Authors:** Luise A. Seeker, Sarah L. Underwood, Rachael V. Wilbourn, Jennifer Dorrens, Hannah Froy, Rebecca Holland, Joanna J. Ilska, Androniki Psifidi, Ainsley Bagnall, Bruce Whitelaw, Mike Coffey, Georgios Banos, Daniel H. Nussey

**Affiliations:** 1 Animal & Veterinary Sciences, SRUC, Roslin Institute Building, Easter Bush, Midlothian, UK; 2 Centre for Regenerative Medicine, Institute for Regeneration and Repair, The University of Edinburgh, Edinburgh BioQuarter, Edinburgh, UK; 3 Institute of Evolutionary Biology, School of Biological Sciences, University of Edinburgh, Edinburgh, UK; 4 Centre for Biodiversity Dynamics, NTNU Norwegian University of Science and Technology, Trondheim, Norway; 5 The Roslin Institute and Royal (Dick) School of Veterinary Studies, University of Edinburgh, Easter Bush, Midlothian, UK; 6 Royal Veterinary College, University of London, Hatfield, UK; 7 SRUC Crichton Royal Farm, Glencaple Road, Dumfries, UK

**Keywords:** Ageing, Senescence, Animal breeding

## Abstract

Telomere length is predictive of adult health and survival across vertebrate species. However, we currently do not know whether such associations result from among-individual differences in telomere length determined genetically or by early-life environmental conditions, or from differences in the rate of telomere attrition over the course of life that might be affected by environmental conditions. Here, we measured relative leukocyte telomere length (RLTL) multiple times across the entire lifespan of dairy cattle in a research population that is closely monitored for health and milk production and where individuals are predominantly culled in response to health issues. Animals varied in their change in RLTL between subsequent measurements and RLTL shortened more during early life and following hotter summers which are known to cause heat stress in dairy cows. The average amount of telomere attrition calculated over multiple repeat samples of individuals predicted a shorter productive lifespan, suggesting a link between telomere loss and health. TL attrition was a better predictor of when an animal was culled than their average TL or the previously for this population reported significant TL at the age of 1 year. Our present results support the hypothesis that TL is a flexible trait that is affected by environmental factors and that telomere attrition is linked to animal health and survival traits. Change in telomere length may represent a useful biomarker in animal welfare studies.

## Introduction

Telomeres are repetitive DNA sequences that cap the ends of eukaryote linear chromosomes^1,2^. They shorten with the number of cell divisions in vitro as well as in response to oxidative stress and critically short telomeres trigger a DNA damage response that leads to replicative senescence or apoptosis^3–5^. In the last decade or so, measures of average telomere length (TL) taken from blood samples have emerged as an exciting biomarker of health across disciplines including biomedicine, epidemiology, ecology and evolutionary biology^6–8^. Considerable among- and within-individual variation in TL has been observed, with a general pattern of rapid telomere attrition during early life and a plateau or slower decline thereafter^9,10^. Both genetic and environmental factors, particularly those associated with physiological stress, predict TL in humans and other vertebrates^11–16^. TL has also been repeatedly associated with health outcomes and subsequent survival in a variety of species, particularly humans and birds^7,17^ and experimentally elongated TL in mice was associated with a survival advantage^18^. However, a major outstanding question remains to what degree associations between TL and health arise from constitutive differences in TL among individuals set by genes or early life conditions^19^, or from the pattern of within-individual change in TL across individuals’ lives which may arise in response to environmental stressors^16^.

Estimates of the individual consistency of TL over time in both the human and avian literature vary considerably among studies. Some studies report very high intra-individual correlations, repeatability measures or heritability measures^19–23^ which indicate that the rank order in TL among individuals may remain relatively consistent over time^19^. This implies most of the variation in blood cell TL occurs at the among-individual level and is predominantly determined by genetics and early-life environment^19,22^. In stark contrast to this, a growing body of literature studying both humans and non-human vertebrates reports much lower individual repeatability and heritability^14,24^. These studies demonstrate very high levels of within-individual variation in TL and show that changes in TL across consecutive measurements are highly dynamic^25,26^. Although on average TL attrition over time tends to be the norm, a growing number of studies have found that a substantial proportion of individuals show telomere elongation over time^26–35^. There is some evidence from cross-sectional studies that in avian and mammalian species a stronger negative correlation between TL and age is associated with a shorter maximum recorded lifespan^36,37^. However, those studies did not test if within a species more telomere attrition predicts shorter individual lifespan. To date, few studies have directly compared the relative power of an individual’s average or early life TL versus the pattern of within-individual change in TL to explain variation in measured TL associated with health and fitness. In Seychelle warblers and Alpine swifts both telomere length and telomere attrition rate were associated with survival^27,38^ while in jackdaw and white‐browed sparrow‐weaver nestlings early life telomere attrition predicted lifespan^20,39^. However, in a study on elderly humans, there was an association between telomere length and longevity, but not between telomere attrition and life span^40^.

Correlative studies in humans and experimental studies mostly in birds show that stressors such as a poorer socio-economic status^13^ or increased caring responsibilities in humans^15^ or an increased reproductive effort in birds^41^ are associated with shorter TL or faster telomere attrition which has also been shown in a recent meta-analysis on non-human vertebrates^16^. Interestingly external factors such as weather have recently also been shown to correlate with telomere dynamics: In the bat species *Myotis myotis*, harsher weather conditions during spring correlated with more attrition in TL^42^ and in a cross-sectional study American black bears living at higher latitudes and thus colder weather had shorter telomeres^43^. Birth cohort effects on TL in longitudinal studies on Soay sheep and European badgers may also reflect environmental factors that may include yearly weather variation^25,44^.

Here, we use an exceptionally detailed longitudinal study of dairy cattle to test which intrinsic and extrinsic factors are associated with change in relative leukocyte telomere length (RLTL), and how average RLTL and telomere attrition predict productive lifespan. We use samples and data collected as part of the long-term study of Holstein Friesian dairy cattle kept at the Crichton Royal Research Farm in Dumfries, Scotland^45^ in association with weather data obtained from a Metoffice station close to the farm. High humidity and temperatures can easily cause heat stress in cattle which leads to changed behaviour such as active seeking of shade and reduced milk productivity^46,47^. We therefore hypothesise that warmer weather may be an extrinsic stressor capable of causing more telomere depletion. At our farm animals of two distinct genetic groups (a group that has been strongly selected for high milk fat and protein yield, and a control group) are randomly allocated to two different diets (a high forage, low energy diet vs. a low forage, high energy diet group)^45^. Blood samples are routinely collected from members of the herd, initially within 15 days after birth and then approximately annually thereafter (Figure S1A,B). Productive lifespan, which is the age of an individual at culling, is recorded for every animal together with a reason for culling which is typically health-related.

Our objectives were to (1) calculate and examine measures that describe lifetime telomere change, (2) test which intrinsic and extrinsic factors are associated with change in RLTL, (3) examine how different measures of telomere change may predict productive lifespan.

## Results

### RLTL profiles and change measurements

**Figure 1:**
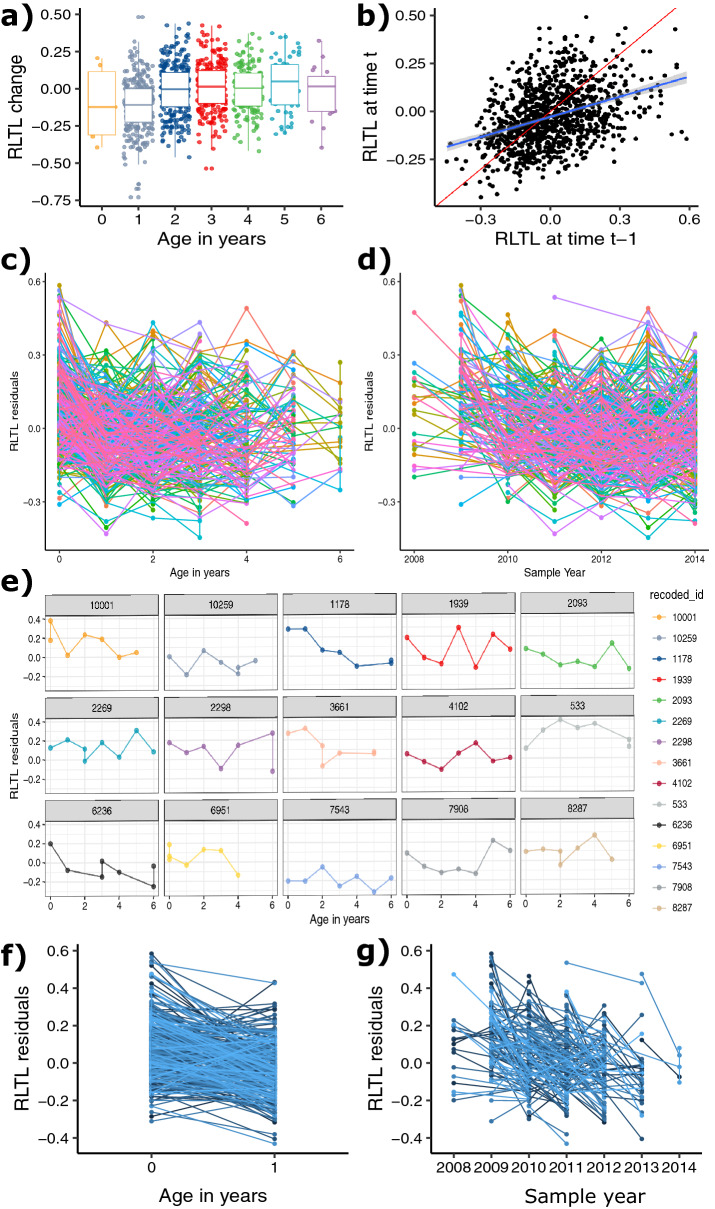
Relative leukocyte telomere length (RLTL) dynamics in dairy cattle. All RLTL measurements were pre-adjusted for qPCR plate and qPCR row to account for known sources of measurement error. (**a**) Age in years at second RLTL measurement is significantly associated with RLTL change. (**b**) At all measurement times the present measurement (RLTL at time t) is clearly correlated with the previous measurement (RLTL at time t − 1) (estimate = 0.38, *P* < 0.001). The red line represents a perfect correlation. (**c**) RLTL over age in years. (**d**) RLTL over sample year. (**e**) Longitudinal RLTL change over age for all animals with at least 7 samples as example for RLTL variation. (**f**) Early-life change in RLTL over age at sampling and (**g**) over sampling year.

We used blood samples collected between 2008 and 2014 to measure longitudinal RLTL by monoplex qPCR in 1,325 samples from 305 female individuals. On average, 4.3 (range: 2–8) telomere measurements were made of each individual, including the first measurement within 15 days of birth and a variable number of subsequent measures (Figure S1C). RLTL measures were adjusted for qPCR plate and row to account for known sources of measurement error^48–50^ and both RLTL change between subsequent measurements (Figure S1D) as well as RLTL residuals (Figure S2A) were approximately normally distributed. The mean of all RLTL change measurements was statistically significantly smaller than zero (*P* < 0.001) indicating that telomere shortening was more frequent than lengthening. Animals varied in the amount and direction of RLTL change across consecutive measurements, with a relatively even proportion of individuals increasing (43.2%) and decreasing (56.8%) in RLTL over time. Figure 1 a and Figure S1D visualise that at young ages RLTL shortens on average, but at older ages RLTL change centres around zero. Consecutive RLTL measurements made on the same individual were overall moderately positively and significantly correlated (r = 0.38, 95% CI: 0.32–0.43, *P* < 0.001; Fig. 1 b), supporting our previously reported moderate and significant individual repeatability of RLTL^50^. In contrast, RLTL change within the individual was not repeatable (repeatability as variance due to the animal divided by the total variance = 0.00) and the repeatability of absolute RLTL change was small (0.049).

Within individuals we observed no constant telomere attrition, maintenance or elongation, but more complex dynamics with short term changes in both directions (Fig. 1c,d). To illustrate this more clearly, example individual RLTL dynamics are shown for all cows with at least seven RLTL measurements in our dataset in Fig. 1e. This complexity means that it is impossible to compare lifetime telomere change dynamics by simply comparing slopes. Therefore, we calculated and examined three average metrics of RLTL dynamics over every individual’s lifetime: Firstly, the average of all their RLTL measures (“mean RLTL”) which investigates if animals with on average longer RLTL have a survival advantage. Secondly, the average of all their RLTL change measures (“mean RLTL change”) and thirdly, the average of all their absolute RLTL change measures (“mean absolute RLTL change”). Mean RLTL averages across the differences between all subsequent RLTL measures, and short-term positive and negative changes can cancel each other out, leaving mostly long-term overall changes to investigate that also consider the overall direction of change. Mean absolute RLTL change, on the other hand, averages across absolute differences between all subsequent RLTL measurements and is used to investigate the hypothesis that RLTL change regardless of direction (meaning the amplitude of change) may be associated with a negative health outcome. Figure S3 offers additional visual explanation.

We were also interested in investigating, if early life telomere dynamics were a predictor of productive lifespan (Figure S2D), similarly to what has been observed in bird studies before^20,39^, because early life predictive measures are of particular interest to the dairy industry. Early life telomere dynamics differ from later telomere dynamics in dairy cattle in that there is more consistent and obvious telomere shortening observed during that time. We focussed on RLTL change within the first year of life by only considering two RLTL measurements per animal: The first was taken shortly after birth and the second at the approximate age of one year (Figure S4A), but because calves are born throughout the year and the second sample is usually taken during an annual sampling in spring, there is some variation in sampling interval (Figure S4b). We calculated RLTL change between those two measurements and observed that most animals (76%) experienced shortening of RLTL within their first year of life (Fig. 1f,g, Figure S4D–F). Sampling interval does not correlate with change in RLTL (r = 0.008, 95% CI = − 0.107–0.123, *P* = 0.891; Figure S5).

### Factors associated with change in RLTL

We next ran a series of statistical model analyses to test whether known individual, genetic and environmental variables could explain variation in RLTL change. Only sampling year was statistically significant in the initial full model; age in years, genetic group, feed group, birth year, the time difference between sample dates in days, and the occurrence of a health event within two weeks of sampling were not significant (Table S1 & Table S2). Genetic group, feed group, and the time interval between sampling were kept in the model to capture the structure of the experiment, but all other non-significant effects were backwards eliminated. In the reduced model age in years was statistically significant with older animals showing less telomere depletion (0.026 ± 0.006, *P* < 0.001; Fig. 1a, Table S3 & Table S4). This model was used to calculate the repeatability of telomere change and absolute telomere change as the variance due to the animal divided by the total variance. We hypothesised that milk production (Figure S2C) may affect change in telomere length, but found no statistically significant relationship between average lifetime milk productivity and change in RLTL, when tested in a subset of animals that had milk productivity measurements available (253 animals with 918 RLTL change measurements, Table S5 & Table S6). We have previously shown that, on average across this population, RLTL declined over the first year of life but showed no systematic change with age thereafter^49,50^. Consistent with this, we found that average RLTL change across consecutive measurements was only significantly negative (indicating a tendency for attrition over time) when the first measurement was made close to birth and the follow up measurement at the age of around 1 year (Fig. 1a, − 0.115 ± 0.01, *P* < 0.001; Table S7).

**Figure 2:**
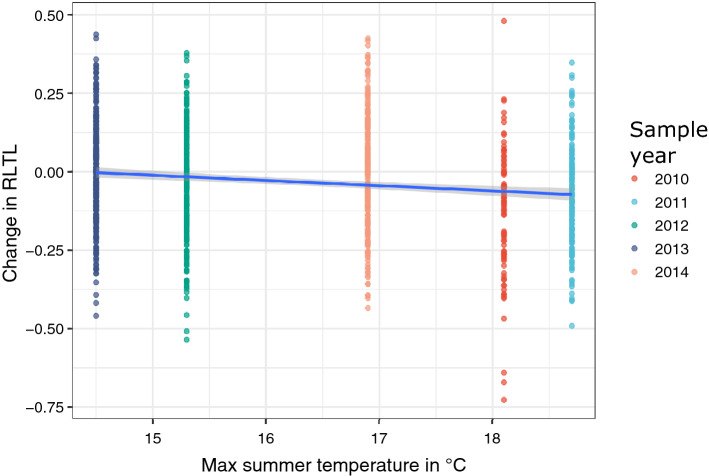
Association between maximum summer temperature and RLTL attrition.

We observed an association between sample year and change in RLTL (F = 3.84, df = 4, *P* = 0.004, Table S4) and hypothesised that this might be at least partially due to different weather conditions. We therefore used weather data (Figure S6) from a Met Office station close to the farm to test if maximum temperature, minimum temperature, average sun hours per day, total rainfall (mm), and total air frost days in the summer and winter quarters correlated with change in RLTL. Maximum temperature over the summer quarter was statistically significantly and negatively correlated with change in RLTL (− 0.012 ± 0.004, *P* = 0.001, Fig. 2) meaning that we observed more RLTL attrition in hotter summers (Table S8 & Table S9). When sample year was included in the same model with maximum summer temperature it became non-significant, while maximum summer temperature remained statistically significant (Table S10 & Table S11), indicating that summer temperature may be the reason for observed yearly variation in RLTL change. The total number of sun hours averaged across the summer quarter (as another marker for a hot summer) was also negatively correlated with change in RLTL (− 0.001 ± 0.000, *P* = 0.021, Table S12 & Table S13) although the effect size was smaller. Rain during summer may contribute to cool down animals and therefore alleviate RLTL attrition, but in our study population where half of the animals are housed continuously it has a marginal effect (0.000 ± 0.000, *P* = 0.041; Table S14 & Table S15). Interestingly, maximum winter temperature was also negatively correlated with change in RLTL (− 0.014 ± 0.005, *P* = 0.009; Table S16 & Table S17), however, when fitted together with maximum summer temperature, it became non-significant (Table S18 & Table S19).

After having observed a summer temperature effect on telomere length dynamics, we were interested to find out if similar effects influenced early life telomere length dynamics. We investigated if the amount of early-life RLTL attrition varied with sample year while accounting for the sampling interval in a linear model but did not find any indication for a statistically significant relationship (N = 291, *P* = 0.666). Therefore, we saw no justification for testing the effect of weather variables on early life RLTL attrition.

We next were interested to find out if there were factors that could predict lifetime RLTL change in the complete dataset and considered the total number of specific disease events as internal stressors. More specifically we looked first at the effect of the number of mastitis and lameness events and then at the number of accumulated mastitis and lameness events together (Table S7) on mean RLTL, mean RLTL change and mean absolute RLTL change, but found no statistically significant relationships (Table S20).

### RLTL change and productive lifespan

**Figure 3:**
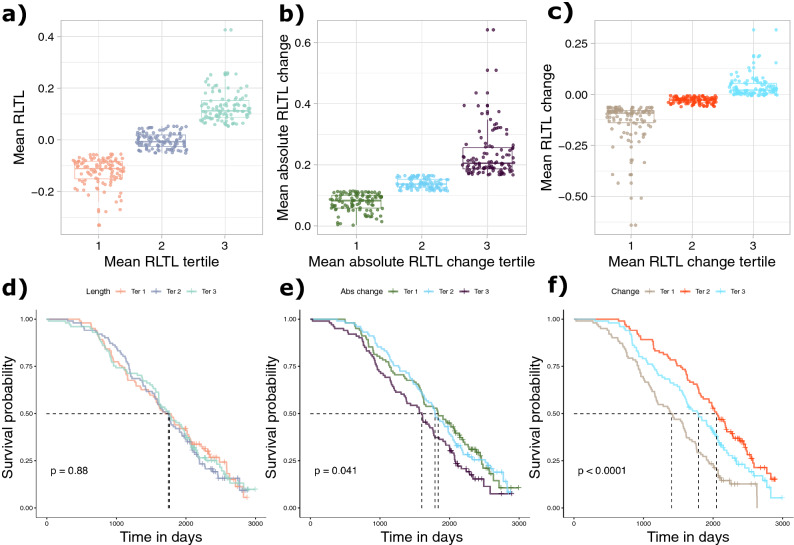
Relationship between lifetime telomere length dynamics and productive lifespan. RLTL measurements were grouped into tertiles as shown for (**a**) mean RLTL, (**b**) mean absolute RLTL change and (**c**) mean RLTL change. Kaplan–Meier curves show relationship of lifetime RLTL dynamics tertiles with productive lifespan. (**d**) Greater lifetime mean RLTL (tertile 3) was not significantly differently associated with productive lifespan than moderate or short lifetime mean RLTL (tertiles 2 and 1). (**e**) Greater mean absolute RLTL change (tertile 3) measured over the lifetime was associated with shorter productive lifespan compared to animals with moderate (tertile 2) or little (tertile 1) mean absolute RLTL change. (**f**) Greater mean lifetime RLTL attrition was associated with shorter productive lifespan. It can be seen that the mean survival of the group with the most RLTL attrition was about 600 days shorter than the mean survival of the group showing the most stable RLTL with neither dramatic shortening nor elongation (tertile 2).

Of all dead cows (N = 244) the vast majority (N = 241) had survived to their first lactation, but there was considerable variation in productive lifespan beyond this point (Figure S2D). We wanted to find out if the three measures of life-long change in RLTL (mean RLTL, mean RLTL change and mean absolute RLTL change) could predict productive lifespan and tested them first separately, then together in the same Cox proportional hazard model. Both mean RLTL change (− 5.209 ± 0.845, *P* < 0.001; Table S21) and mean absolute RLTL change (2.939 ± 0.970, *P* = 0.002; Table S21) were significantly associated with productive lifespan while mean RLTL was not (coefficient = 0.341, SE = 0.591, *P* = 0.564, Table S21). When all three measures of lifetime RLTL dynamics were included in the same model only mean RLTL change remained significant (− 4.758 ± 1.018, *P* < 0.001; Table S21). This implies that the relationship between productive lifespan and mean absolute RLTL change was largely due to covariance with mean RLTL change. Thus, individuals that experienced greater telomere attrition over their lifetimes had a shorter productive lifespan and direction of RLTL change (rather than simply absolute magnitude) was an important aspect of this relationship. To visualise the association between RLTL change measurements and productive lifespan using Kaplan–Meier plots, continuous RLTL measures were transformed to a discrete scale by grouping them into tertiles (Fig. 3). Cox proportional hazard models based on these tertile groupings of RLTL measures showed similar results to those reported above (mean RLTL: 0.014 ± 0.079, *P* = 0.858; mean RLTL change: − 0.257 ± 0.087, *P* = 0.003, mean absolute RLTL change: 0.179 ± 0.082, *P* = 0.029, Table S22).

The relationship between mean RLTL change and productive lifespan was robust to the inclusion of milk production (a physiological stressor, which is positively associated with productive lifespan as cows with a low milk yield are more likely to be culled at a younger age) in the model (Table S23). If RLTL declines mostly within the first year of life (as Fig. 1a indicates), the association between change in RLTL and productive lifespan may be driven by the fact that this initial decline contributes relatively more to estimates of mean RLTL change in shorter-lived individuals than in animals that have more follow up samples with more moderate change measures available. We therefore repeated the analysis excluding the early life RLTL change measurements and found that mean RLTL change still predicted productive lifespan (N = 253, coefficient = − 5.056, SE = 1.315, *P* < 0.001, Table S24). The relationship between mean RLTL change and productive lifespan also remained statistically significant, when animals with fewer than 3 samples (which may be more affected by outlier measurements) were excluded from the analysis (− 3.47 ± 1.34, N = 213, Wald test = 6.7 on 1 df, *P* = 0.01). In our previous studies we thoroughly tested RLTL at different ages as a predictor of productive lifespan and found that while RLTL at the age of one year correlated with survival, RLTL at other ages (including at birth) did not^50^. In the present study we found that mean RLTL change was a better predictor of productive lifespan than RLTL at the age of one year when tested in the same model (Table S25).

Most reasons for culling in our herd were disease-related, but some reasons included accidents and herd management procedures and for some animals the reason for culling remained unknown (Figure S8). Even when animals without a recorded disease-related reason for culling were excluded from the analysis, mean RLTL change still predicted productive lifespan (Table S26).

**Figure 4:**
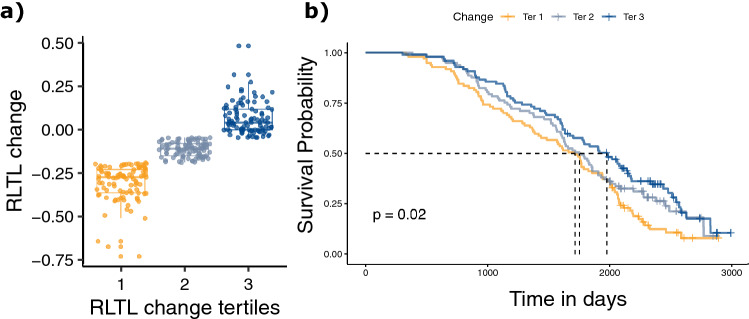
Early-life change in RLTL and productive lifespan. (**a**) visualises how continuous RLTL change measurements were grouped into discrete tertiles to allow visualising of survival data. (**b**) Kaplan–Meier plot that shows the association of early- life RLTL change tertiles with productive lifespan. More RLTL attrition within the first year of life (tertile 1) was associated with a shorter productive lifespan.

Similarly, we used a Cox proportional hazard model to test whether early life RLTL change between two samples, one taken shortly after birth and the next at an approximate age of one year, predicted productive lifespan and found that greater early life RLTL attrition was associated with a shorter productive lifespan (− 1.141 ± 0.391, N = 291, *P* = 0.004, Table S21). When we repeated the analysis using the discrete measure of RLTL change tertiles for visualisation purposes we obtained similar results (− 0.225 ± 0.082, N = 291, *P* = 0.006, Fig. 4, Table S22). In parallel to the analysis of the whole dataset, the relationship between early life change in RLTL remained statistically significant, when animals without a recorded health-related reason for culling were excluded (Table S26).

## Discussion

Our study animals varied considerably in the magnitude and direction of RLTL change across consecutive sampling points. This is in accordance with other longitudinal studies that have reported a wide variation in TL change, alongside observations that a large proportion of individuals actually exhibit telomere lengthening over time^26–35^. Previous work has suggested that rapid changes observed in telomere length, and particularly apparent telomere lengthening may be due to measurement error affecting mostly qPCR results^51^. However, other studies using simulated data have also shown that telomere lengthening might be biological and not solely due to measurement error^52,53^. For the present study we carefully optimised the qPCR protocol to ensure reproducible results that are also robust to extracting DNA repeatedly from the sample using different DNA extraction techniques^48^. Our qPCR measurements are repeatable: the proportion of total variance due to sample variance is 80%, consecutive measures correlate well (Fig. 1b) and baseline measurements do not correlate strongly with future mean rate of RLTL changing rate (Figure S9) which has previously been used as a marker for a small measurement error^51,54^. This makes us confident that our measurements overall capture biological variation. We show that, despite TL being moderately consistent across the lifetimes of individuals, considerable within-individual variation exists and the pattern of change in TL over an individual’s life is highly dynamic. Short-term environmental fluctuations impacting TL dynamics could be responsible and may impact individuals in different ways. A recent meta-analysis has shown that different kinds of stressors are associated with telomere loss in non-human vertebrates^16^. We aimed to understand factors influencing telomere change in our study system and found that age was associated with change in RLTL in the following way: young animals on average shortened their TL, but older animals did not show a systematic relationship of change in RLTL with age. This is in accordance with our previous cross-sectional observations in this study population^49,50^. We further found that sample year was associated with change in RLTL and hypothesised that the yearly effect may be partially explained by weather variables after similar observation have been made for other species: In bats stressful weather conditions during a critical time of the year was associated with more telomere attrition^42^. Dairy cattle are metabolically incredibly active and therefore easily experience heat stress in warm and humid climates^46,47^. Dairy cows actively seek shade when temperatures are above their comfort range^47^, which is a behaviour frequently observed on our research farm in Dumfries during the summer months. Indeed, we found using data from a weather station located close to the farm that during hot summers animals experienced more RLTL attrition. Our results indicate that organismal stress is associated with more telomere depletion and thus provide first evidence that change in telomere length may indeed be useful as a biomarker for animal welfare in farm animals as suggested before^55^. In the specific case of heat stress, it is likely that more easily accessible measures such as milk productivity^56^ will be more helpful on commercial farms. Our observation that weather correlates with telomere dynamics supports previous findings that TL is affected by environmental conditions^12,16,42^. We could not find evidence in our study that the number of fertility and mastitis events (investigated separately and together) correlated with life-long RLTL change measures. A reason for this may be the crude categorisation of those disease events and adding severity scores in future analyses may influence the result.

Individuals with a greater propensity to lose TL over time in our study had shorter productive lives, implying changes in TL reflect important environmental or physiological variation linked to health. We have previously shown that there is a genetic correlation between RLTL at birth and productive lifespan indicating that genes for long telomeres and genes for an improved productive lifespan may be in linkage disequilibrium and inherited together^57^ or pleiotropic genes causing long telomeres also improve survival chances. Our data support the contention that within-individual directional change over time in TL is more important than among-individual differences in predicting overall health. While our results that early life attrition in TL correlates with lifespan is in accordance with several bird studies that reported similar results^20,39^, the present study is to our knowledge first demonstration that lifetime variation in telomere attrition rather than variation in constitutive individual differences in average TL predict health outcomes and lifespan in any vertebrate. While there is mounting evidence that TL predicts mortality, health and life history in humans as well as birds and non-human mammals^7,17,23–25,57–62^, very few studies have been able to accumulate long-term longitudinal data capable of differentiating the role of among- and within-individual variation in TL to such relationships. There was no relationship between productive lifespan and an individual’s average RLTL in the present study.

Future studies will show how well our results generalise to other systems as telomere biology is variable amongst species. Cattle telomere biology seems to be similar to other ruminants, horses, zebras, tapirs, some whales and primates including humans in that they have relatively short telomeres and a tight regulation of telomerase expression^63^. If our results extend to some of those other systems and contexts, they have important implications for the utility of TL as a biomarker of health and fitness, lending support to the idea that change in TL is an indirect marker reflecting past physiological insults and stress rather than an indicator of constitutive or genetically-based robustness to life’s challenges. Our data also highlight the importance of collecting longitudinal telomere measurements, by showing that in some species it is within-individual change over time in TL that carries the important biological signal.

## Materials and methods

We aimed to follow ARRIVE guidelines^64^ throughout this manuscript and provide the ARRIVE essential information in Supplementary File 3.

### Animal population and data collection

We used samples and data collected as part of the long-term study of Holstein Friesian dairy cattle kept at the SRUC Crichton Royal Research Farm in Dumfries, Scotland^45^. This herd, consisting of around 200 milking cows plus their calves and replacement heifers, has been regularly monitored since 1973 for a broad range of measurements, such as body weight, feed intake, signs of disease (health events), milk yield, productive lifespan and reasons for culling^45^. One half of the milking cows belong to a genetic line that has been selected for high milk protein and fat yield (S), while the other half is deliberately maintained on a UK average productivity level (C). Calves and heifers of both genetic lines are kept together. After first calving all cows are randomly allocated to a high forage (HF) or low forage (LF) diet. The LF diet is energy richer than the HF diet and whilst the LF cows are housed continuously, the HF cows graze over the summer months. All cows are milked three times daily and milk yield is recorded. In the present study, these measurements were used to calculate an average milk production in kg per cow including all started lactations and it is referred to this as “average lifetime milk production” (Figure S2C). Every day cows leave the milking parlour over a pressure plate which detects signs of lameness. Behaviour and health events are documented after visual detection by farm workers (Figure S7). At the end of the animal’s life its productive lifespan (Figure S2D) and a reason for culling are recorded (Figure S8). Productive lifespan is the time from birth to culling in days and is a proxy for the health span of the animal, because all animals that remain healthy enough to generate profit for the farmer remain in the herd. The most frequent reasons for culling were reproductive problems, mastitis, lameness which are typically the most frequent cull reasons on a commercial dairy farm (Figure S8). Further information about the animal population can be found in Supplementary File 2.

### Blood sampling

We collected 1,325 whole blood samples from 305 female individuals in the years 2008 to 2014. Routine blood sampling takes place initially shortly after birth (within 15 days of birth) and then annually in spring (Figure S1A,B). If possible, an additional sample is taken shortly before an animal is culled. Because of this sampling routine and because calves are being born all year round, age at sampling and sampling intervals vary for animals (Figure S2B, Figure S3B, Figure S4).

### Ethics statement

The SRUC Animal Experimentation Committee approved the blood sampling which was conducted in accordance with UK Home Office regulations (UK Home Office Project License Number: PPL 60/4278 Dairy Systems, Environment and Nutrition).

### Collection of weather data

Weather data was obtained from the Met Office weather station in Eskdalemuir (Location 323400E 602600 N, Lat 55.311 Lon − 3.206, 242 m amsl). Eskdakenuir is with 21.8 miles (35.1 km) direct distance (https://www.freemaptools.com/how-far-is-it-between.htm) the closest weather station to the farm in Dumfries. Weather data included maximum temperature, minimum temperature, days of air frost, total rain in mm and total sun hours for each month (Figure S6). Data was reduced to the years of interest between 2006 and 2015 and summarised to maximise its relevance considering the sampling interval on the farm to quarterly statistics in the following way: Routine blood sampling was performed in March (Figure S1 B) and therefore the calendar year was divided into quarters and then allocated to a “sample year” which ran from April in the previous year to end of March of the year when the blood sample was taken. This ensured that sampling periods for weather and telomere data were synchronised.

### DNA extraction and RLTL measurement

DNA from whole blood samples was extracted with the DNeasy Blood and Tissue spin column kit (QIAGEN) and telomere length was measured by qPCR as previously described^48–50,57^. The repeatability of the assay (see Supplementary File 2 for how repeatability was calculated) was 80% and therefore delivers interpretable results^65^. A full description of our DNA extraction and qPCR protocols including quality control steps can also be found in Supplementary File 2.

### Statistical analysis

All statistical analyses were performed in R studio^66^ with R 4.0.2.^67^. Mixed-effects models were implemented using the ‘lme4’ library^68^, while Cox proportional hazard models were implemented using the library survival^69^ and figures were generated with the library ‘ggplot2’^70^. All statistical packages used and a full description of the analysis including code can be found on GitHub (https://github.com/LASeeker/TelomereChangeInDairyCattle).

#### Accounting for known sources of measurement error

We have shown before that our RLTL data are significantly affected by qPCR plate and qPCR row^48–50^. To account for those known sources of measurement error, we used the residuals of a linear model that corrected all RLTL measurements for qPCR plate and row, by fitting plate and row as fixed factors in the model. These residual RLTL measures were used in all subsequent calculations and models of telomere dynamics.

#### RLTL profiles and change measurements

We calculated 1020 RLTL change measurements of 305 female animals as the difference between two subsequent adjusted RLTL measurements within individual (RLTL change = RLTL_t_-RLTL_t−1_). We used those longitudinal RLTL change measurements as response variables to investigate the impact of various effects such as age, health events and weather conditions on telomere change (see below).

We calculated following three measures of lifetime RLTL change: The animal’s mean RLTL over all measurements, the mean RLTL change and the mean absolute RLTL change. While mean RLTL change captures the direction and magnitude of RLTL changes, mean absolute RLTL change describes just the magnitude of change without considering its direction because we were interested to investigate if more change in either direction may be correlated with adverse effects. Figure S3 visualises the reasoning behind calculating these three measures of lifetime telomere length dynamics which are not surprisingly moderately correlated with one another (r ranged from -0.53 to 0.30, Figure S10).

We were also interested in analysing early life RLTL dynamics and its association with productive lifespan. Therefore, we calculated change in RLTL within the first year of life as the difference between one measurement taken shortly after birth and the next taken at around one year of age (Figure S4A).

#### Factors associated with change in RLTL

To investigate which factors correlate with the direction and amount of RLTL change, a linear mixed model was fitted with RLTL change between two consecutive measurements as response variable and animal identity as random effect. The following factors were included as fixed effects: genetic line, feed group and birth year of the animal, age at sampling (at time t), sample year, and the occurrence of a health event within two weeks before or after sampling (at time t). The time difference between consecutive samplings in days was fitted as a covariate. Non-significant fixed effects (*P* > 0.05) were backwards eliminated from the model. Age at sampling was modelled as a covariate (age in years). We hypothesised that the high metabolic demand of milk production may impact change in telomere length and therefore repeated the above model for a subset of 918 RLTL measurements of 253 animals with a known average lifetime milk production which was fitted as additional covariate. Average lifetime milk productivity was re-scaled by dividing it by 1000 to adjust it to a comparable scale as the other parameters in the model.

We hypothesised that yearly variation in RLTL may be due to yearly variation in weather variables (Figure S6) and re-ran the model above for the whole dataset while including quarterly weather variables as covariates as a replacement for sample year. We restricted weather observations to the summer and winter quarter to capture the most extreme seasons. Following variables were tested: maximum temperature, minimum temperature, total number of air frost days, totals sun hours averaged across the quarter, average rain in mm.

To better understand variables that correlate with early life RLTL, we tested if the amount of early life RLTL attrition (RLTL at 1 year - RLTL at birth) varied with sample year while accounting for the sampling interval in a linear model.

After finding factors that influenced RLTL change, we thought that the accumulated number of specific health events that are associated with inflammation and pain may correlate with lifetime RLTL dynamics (mean RLTL, mean RLTL change and mean absolute RLTL change) and tested our hypothesis using the number of mastitis or lameness events in a linear model; these analyses were run first separately by condition and then collectively by summing all events per animal.

#### Association between lifetime RLTL measures and productive lifespan

We used Cox proportional hazard models of productive lifespan that also included mean milk production as covariate and the three measures of lifetime RLTL dynamics (mean RLTL, mean RLTL change and mean absolute RLTL change) as explanatory variables first separately and then together in the same model to test their association with productive lifespan first individually, then while accounting for the effect of the other two measures. For visualisation purposes we converted the continuous measures of lifetime telomere measures to a discrete scale by using tertiles and repeated the Cox proportional hazard models with those and visualised the relationship in Kaplan–Meier plots.

To ensure observed associations between RLTL change and productive lifespan were not simply due to more rapid RLTL attrition early in life, the initial Cox proportional hazard models (that included RLTL measures on a continuous scale) were repeated first while all measurements that were taken shortly after birth were excluded and then while all animals with fewer than three RLTL measurements were excluded. Additionally, we wanted to better understand if telomere change or telomere length is the better predictor for productive lifespan. We therefore tested if the previously reported effect of RLTL at a specific age (one year) on productive lifespan^50^ remained statistically significant when tested in a Cox proportional hazard model together with milk productivity and mean RLTL change. Lastly, we considered that most, but not all of our animals were culled for health-related reasons (Figure S8) and repeated the Cox proportional hazard models of productive lifespan with lifetime RLTL dynamics measures as predictors for a subset of animals that had a recorded health-related reason for culling.

We were interested to find out if telomere attrition within the first year of life was another predictor of productive lifespan and therefore tested it in a Cox proportional hazard model. We transformed the continuous measure of early life RLTL change to a discrete scale by calculating tertiles and repeated the Cox proportional hazard models using the tertiles as explanatory variables in an effort to visualise the relationship between RLTL change and productive lifespan using Kaplan–Meier plots. Finally, we repeated the Cox proportional hazard analysis of early life RLTL change for those animals that had a recorded disease-related reason for culling. See Figure S11 for a visual description of all Cox-proportional hazard models used in this study.

## Supplementary Information

Supplementary Information 1.

Supplementary Information 2.

Supplementary Information 3.
