# Supplementary material for: Telomere attrition rates are associated with weather conditions and predict productive lifespan in dairy cattle
Source: Sci Rep. 2021 Mar 10;11:5589. doi: 10.1038/s41598-021-84984-2 (PMC7970942; doi:10.1038/s41598-021-84984-2)
Supplement: Supplementary file 1 — Supplementary Information 1. [file 41598_2021_84984_MOESM1_ESM.docx]

# Supplementary File 1

## Supplementary Figures and Tables

# Telomere attrition rates are associated with weather conditions and predict productive lifespan in dairy cattle

**Authors & Affiliations:**

Luise A. Seeker^1,2*^, Sarah L. Underwood^3^, Rachael V. Wilbourn^3^, Jennifer Dorrens^3^, Hannah Froy^3,4^, Rebecca Holland^3^, Joanna J. Ilska^1, 5^, Androniki Psifidi^5,6^, Ainsley Bagnall^7^, Bruce Whitelaw^5^, Mike Coffey^1^, Georgios Banos^1, 5^ & Daniel H. Nussey^3^

^1^ Animal & Veterinary Sciences, SRUC, Roslin Institute Building, Easter Bush, Midlothian, UK

^2^ MRC Centre for Regenerative Medicine, University of Edinburgh, Edinburgh, UK

^3^ Institute of Evolutionary Biology, School of Biological Sciences, University of Edinburgh, UK

^4^ Centre for Biodiversity Dynamics, NTNU Norwegian University of Science and Technology, Trondheim, Norway

^5^ The Roslin Institute and Royal (Dick) School of Veterinary Studies, University of Edinburgh, Easter Bush, Midlothian, UK

^6^ Royal Veterinary College, University of London, Hatfield, UK

^7^ SRUC Crichton Royal Farm, Glencaple Road, Dumfries, UK

***Corresponding author:**

Luise A. Seeker

MRC Centre for Regenerative Medicine*,* The University of Edinburgh*,* Edinburgh BioQuarter, 5 Little France Drive, Edinburgh EH16 4UU

[Luise.seeker@ed.ac.uk](mailto:Luise.seeker@ed.ac.uk)

07591133397

## Supplementary Figures


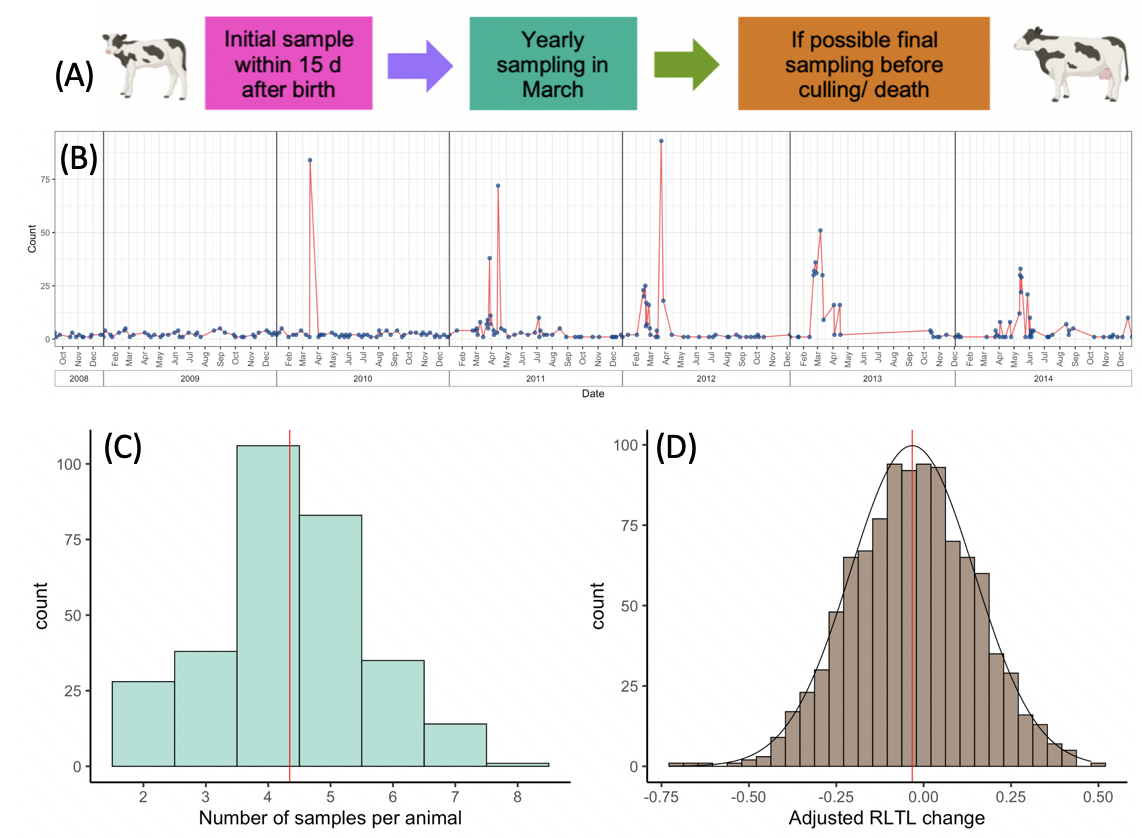


Figure S1: Blood sampling routine for telomere length measurement. (A) Schematic description of sampling protocol (diagram created with BioRender.com). (B) Number of samples taken over sampling date. Spikes represent annual sampling in March. At other times new-born calves and adult animals shortly before culling were sampled. It can be seen that regular sampling in March was established in 2010. (C) Number of RLTL measurements per animal. (D) Distribution of RLTL change after adjusting for qPCR plate and row.


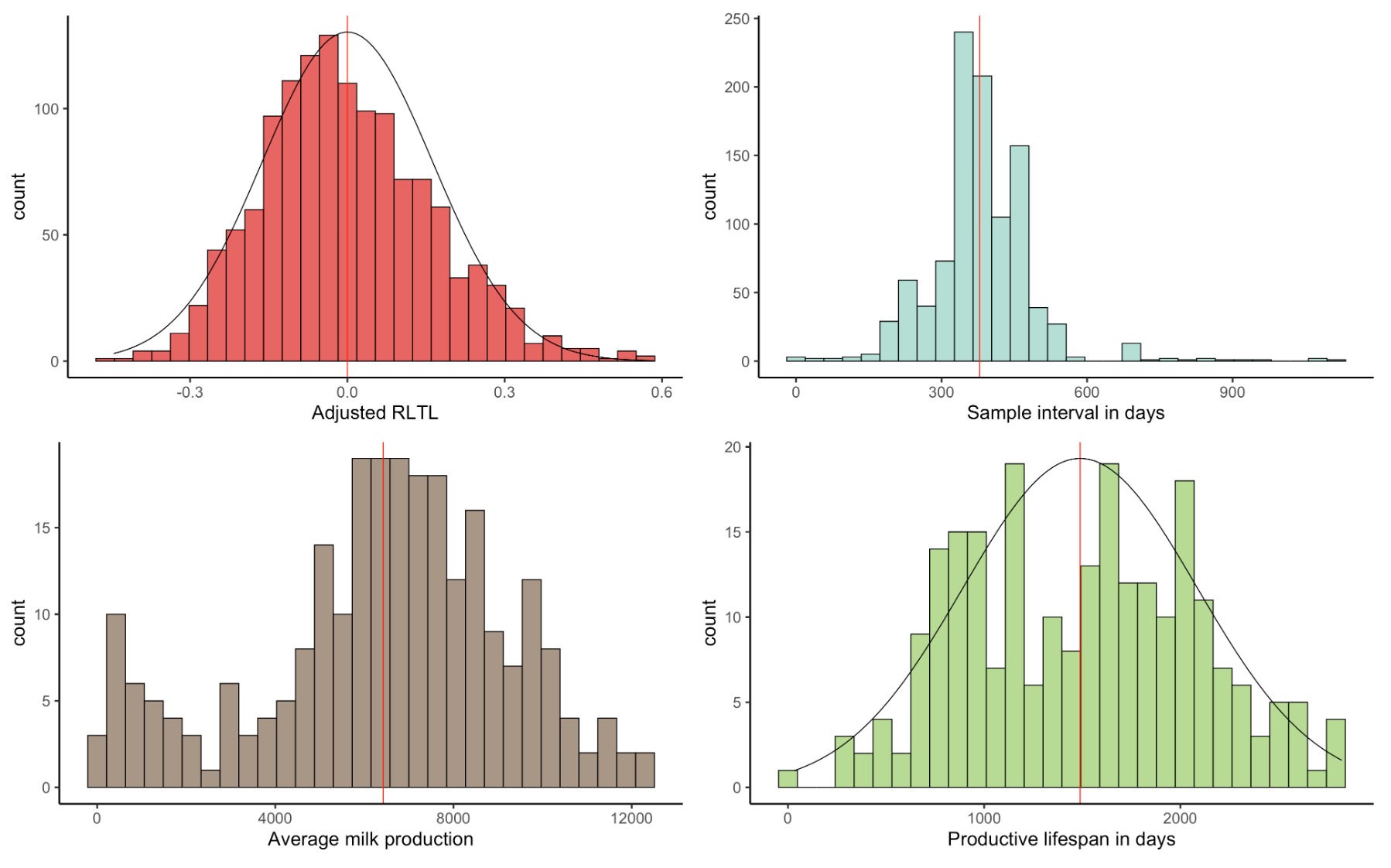


(D)

(C)

(B)

(A)

Figure S2: (A) Distribution of RLTL measurements that were pre-adjusted for qPCR plate and row, two known sources of measurement error. (B) Sample interval between two consecutive samples varied considerably with a mean of approximately one year. (C) Average lifetime milk production for all animals that started at least the first lactation. (D) Distribution of productive lifespan measurements of all animals.


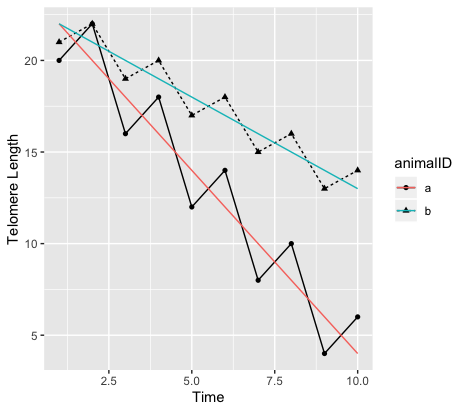

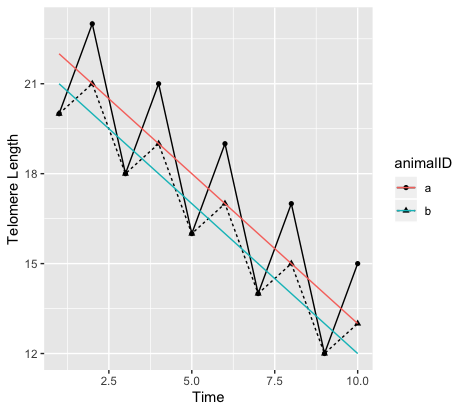

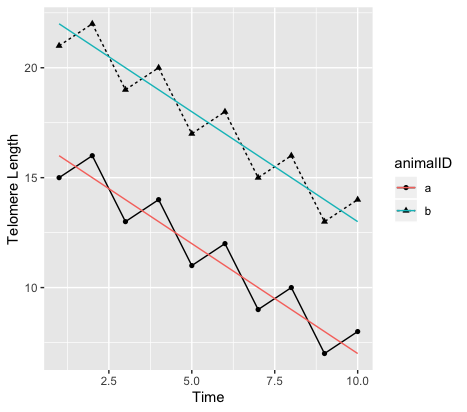


(A)

(B)

(C)

Figure S3: Visualisation of reasoning behind the calculation of different lifetime telomere change measures. Hypothetical scenario (A): differences in mean relative leukocyte telomere length (RLTL). Animals differ in their mean telomere length while telomere attrition rate and variance in telomere length are similar. Animals with longer mean RLTLs over life (for example animal b) are assumed to have a survival advantage over those with on average shorter RLTLs (animal a). Hypothetical scenario (B): Differences in mean absolute RLTL change. Animals do not differ significantly in their telomere length or their overall attrition rate. However, they show a difference in the magnitude of short-term telomere change. It is hypothesised that animals that are able to better maintain their telomere length and show lees absolute telomere change (animal b) have a survival advantage over those that show extreme telomere change in both directions (animal a). Hypothetical scenario (C): Differences in mean RLTL change. Some animals show a faster overall telomere attrition (animal a) compared to others (like animal b) and it is hypothesised that fast telomere attrition is associated with a shorter lifespan.

**
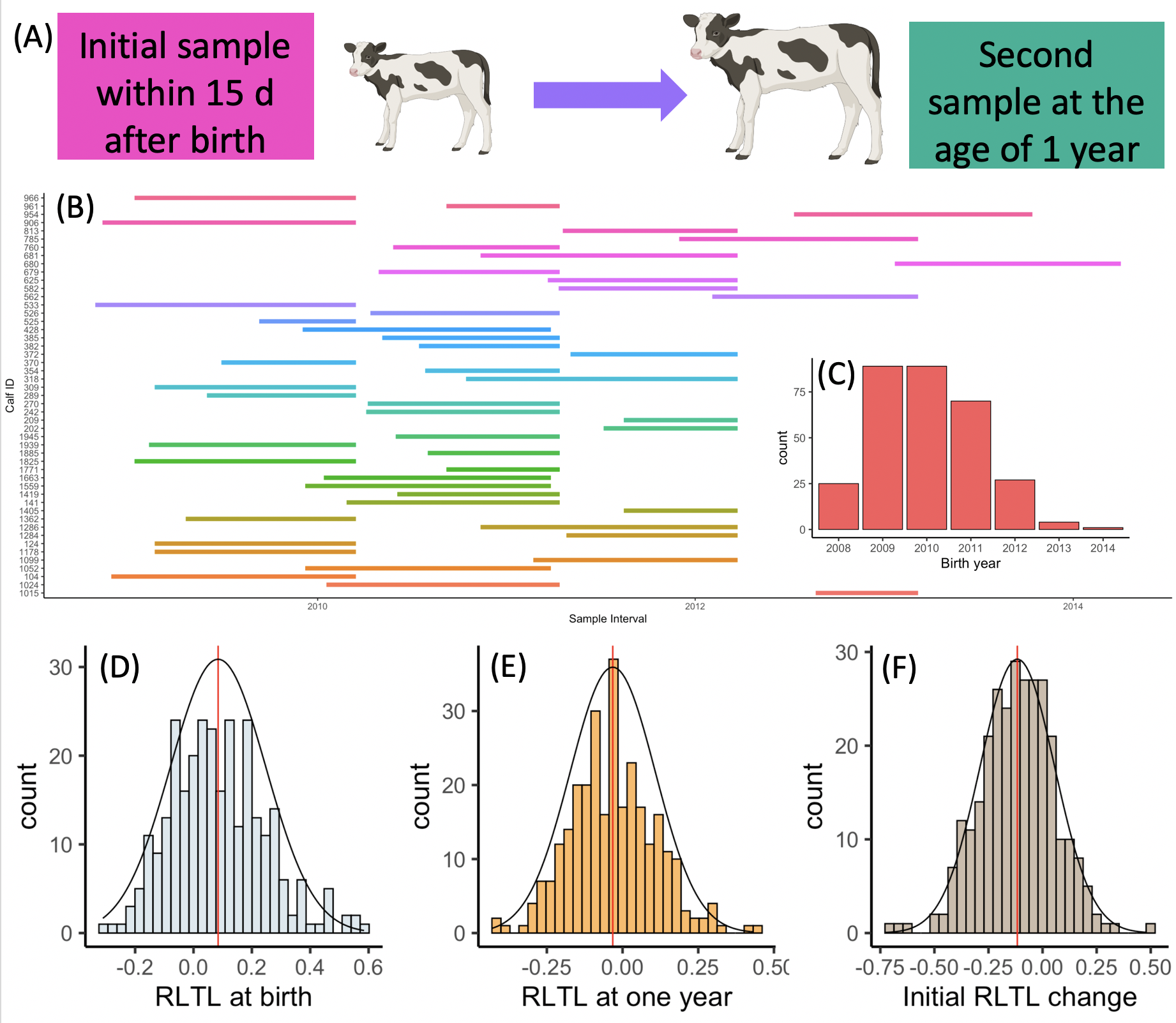
**

Figure S4: Calf dataset. (A) Sampling routine (created with BioRender.com). (B) Representative sampling interval is shown for a subset of 50 randomly chosen animals. (C) Distribution of birth years. (D) Distribution of RLTL at birth and (E) of RLTL close to 1 year of age. (F) Distribution of RLTL change between first two measurements.


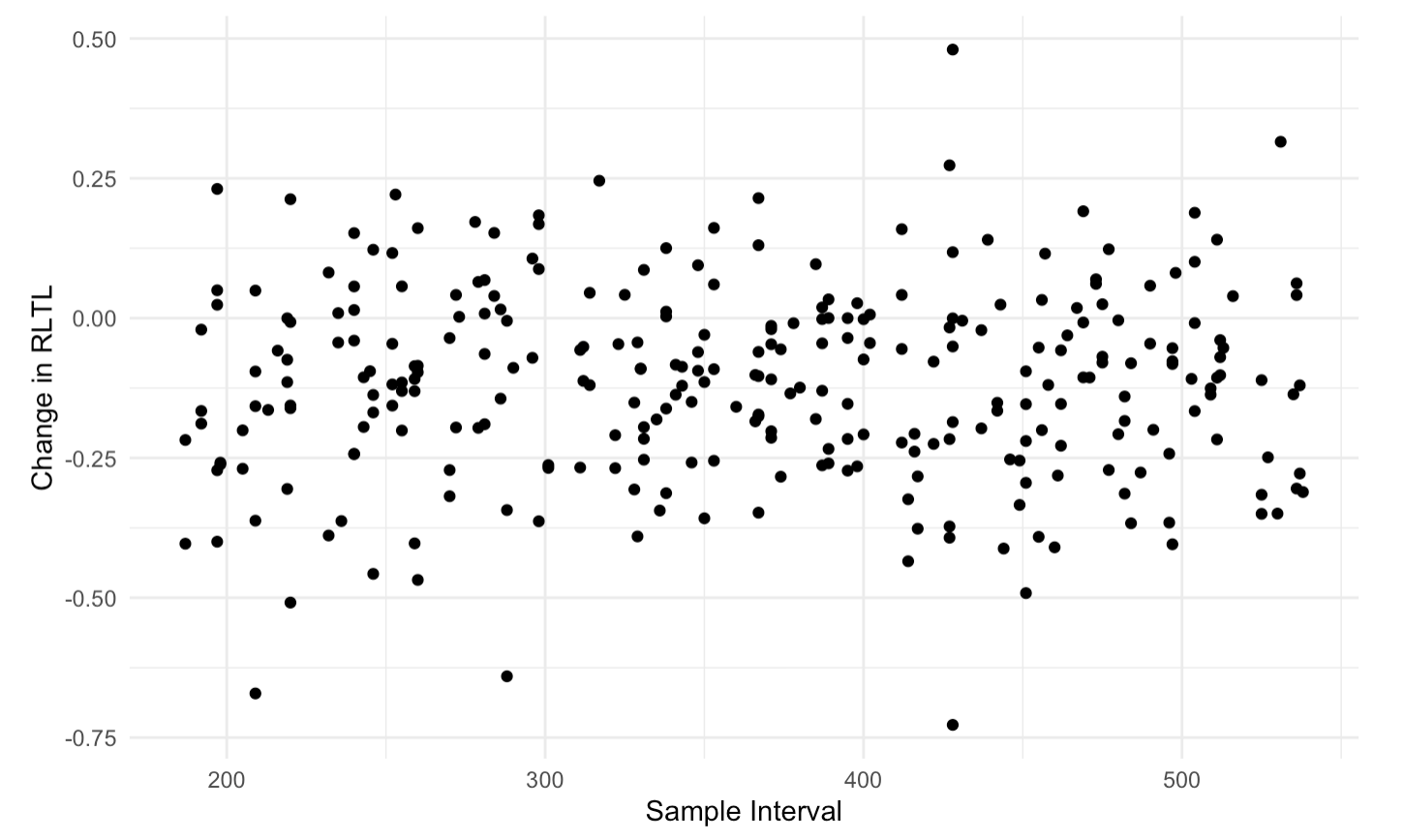


Figure S5: Sample interval does not correlate with change in RLTL (r = 0.008, 95% CI: -0.107 - 0.123, p = 0.892) in early life suggesting that there is variation in the rate of RLTL change.

Figure S6: Weather data obtained from the Met Office station in Eskdalemuir (Location 323400E 602600N, Lat 55.311 Lon -3.206, 242m amsl)


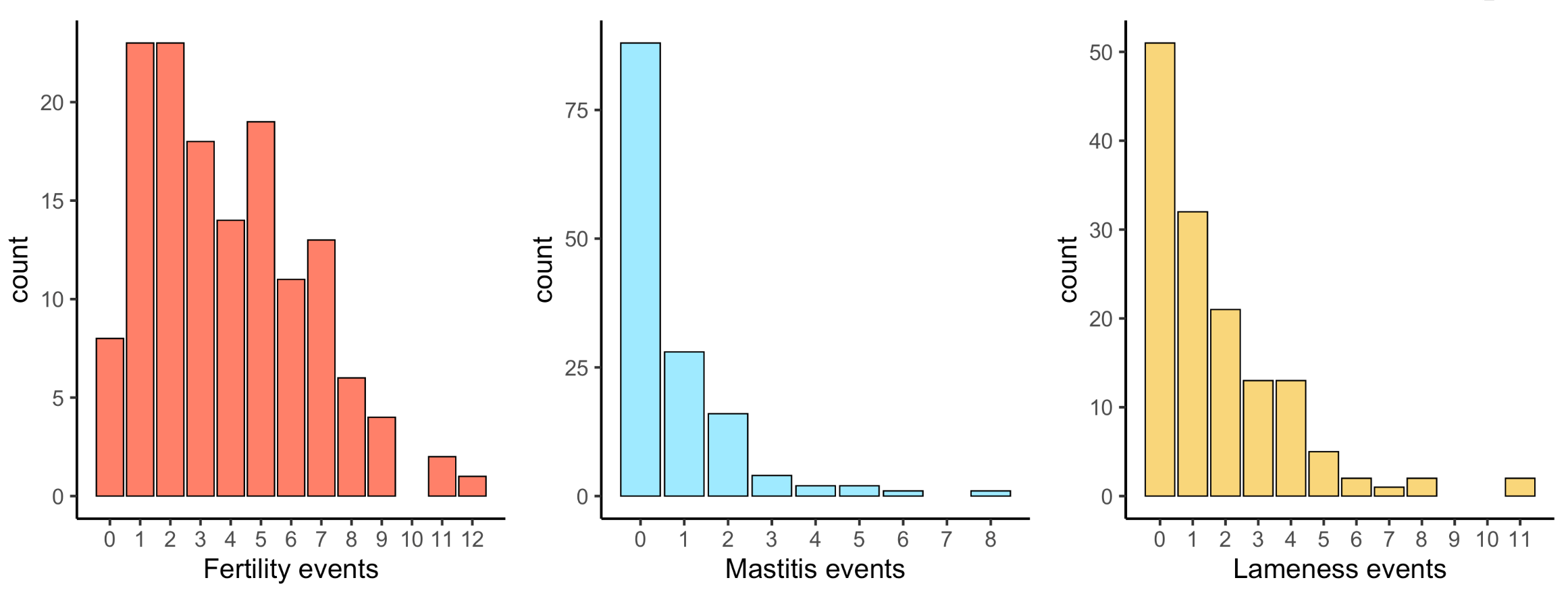


Figure S7: Number of specific health events per animal. While mastitis and lameness events are always pathological, fertility events also include healthy events such as an animal seen to show specific behaviour that indicated ovulation or the event of a calving.

Figure S8: Reasons for culling. Fertility and reproduction, mastitis and lameness are usually the three leading reasons for culling on a dairy farm. They include inflammatory processes that can become systemic. Many diseases affect milk yield and poor milk production can be a sign of an otherwise subclinical disease. Accidents are often associated with bulling behaviour where cows jump onto each other’s back if animals are in oestrus which may lead to bone fractures. Accidents also include downer cows that are trapped in their cubicles and cannot get to their feet. A reason for this may be a metabolic condition called “milk- fever”. Cows removed based on herd management decisions are unlikely to include the healthiest and most productive animals.


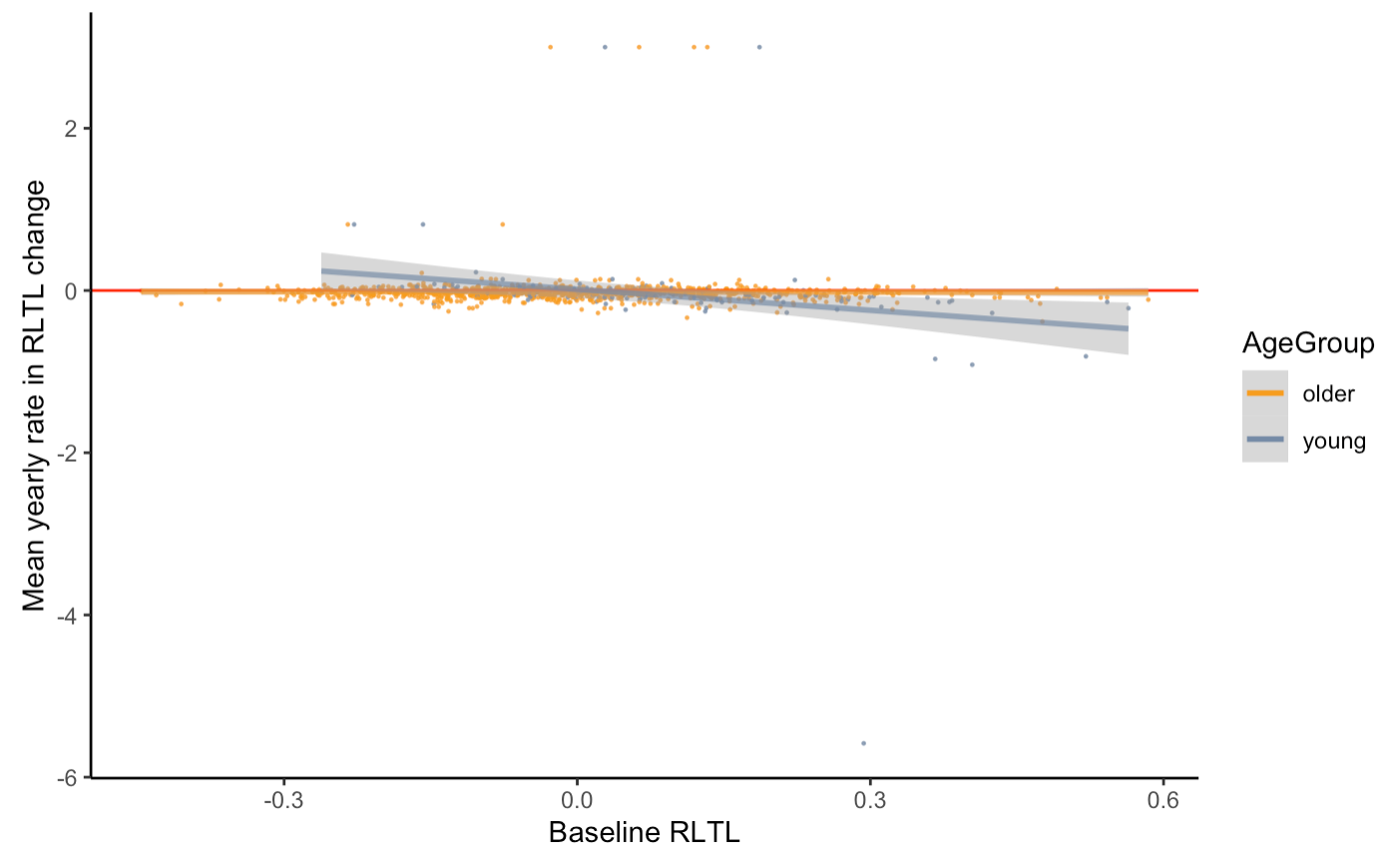


Figure S9: The mean yearly RLTL change rate should not be correlated with baseline RLTL if the measuerement error is. It can be seen that the correlation of those measurements taken during adult years indeed equals zero. Overall there os a slight correlation (r = - 0.07, p = 0.02) which seems to be driven by measurements taken early in life. Since all age groups were measured on the same plates, a biological reason between the correlation of telomere length at birth and lifetime mean telomere attrition rate cannot be excluded.


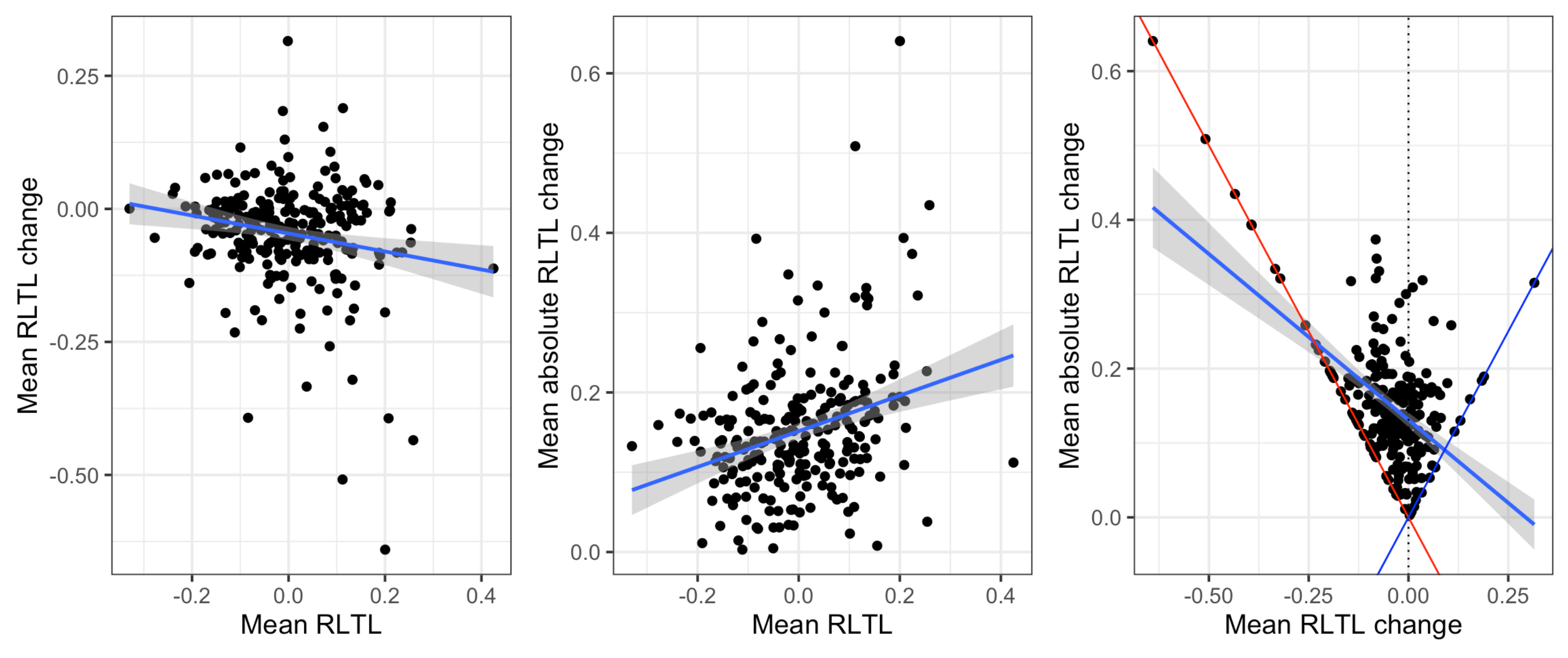


(C)

(B)

(A)

Figure S10: Correlations between three different measures of lifetime relative leukocyte telomere length (RLTL) dynamics calculated for 244 dead animals. (A) Mean RLTL and mean RLTL change: r=-0.18, p=0.004; (B) Mean RLTL and mean absolute RLTL change: r=0.30, p<0.001. (C) Mean RLTL change and mean absolute RLTL change: r=-0.53, p<0.001. The red line represents perfect negative correlation, blue line represents perfect positive correlation. Dotted line represents data that shows a variable amount of absolute change that cancels each other out to result in no overall RLTL change.

Figure S11: Visual description of different models used to investigate telomere length measures as predictors of productive lifespan. Created with Biorender.com. (A) full model containing RLTL measurements of all animals. (B) Early life measures were excluded. (C) Animals with fewer than 3 samples were excluded. (D) The initial model was repeated for animals with milk production records. (E) The previous model (D) was extended by including the previously shown to be significant RLTL measure at the age of 1 year. (F) The initial analysis was repeated for all animals with a recorded health-related reason for culling. (G) The relationship of early life change of RLTL with productive lifespan was tested. (H) Same as (G) but restricted to animals with a recorded health-related reason for culling.

# Supplementary Tables

Table S1: Full initial model. Effect sizes and significance of fixed effects in full linear mixed model of RLTL change, adjusted for qPCR plate and row with animal identity fitted as a random effect.

|  | Estimate | Std. Error | df | t value | p-value |
| --- | --- | --- | --- | --- | --- |
| (Intercept) | -0.126 | 0.039 | 1002 | -3.256 | 0.001 |
| Age in years | 0.022 | 0.014 | 1002 | 1.602 | 0.110 |
| Feed group: high forage (N=110) | -0.008 | 0.012 | 1002 | -0.685 | 0.493 |
| Feed group: not allocated (N= 80) | -0.007 | 0.016 | 1002 | -0.409 | 0.683 |
| Genetic group: select (N=135) | -0.01 | 0.011 | 1002 | -0.896 | 0.371 |
| Birth year: 2009 (N= 90) | -0.014 | 0.021 | 1002 | -0.694 | 0.488 |
| Birth year: 2010 (=89) | -0.012 | 0.029 | 1002 | -0.431 | 0.666 |
| Birth year: 2011 (N=70) | -0.006 | 0.039 | 1002 | -0.164 | 0.870 |
| Birth year: 2012 (N=28) | -0.022 | 0.055 | 1002 | -0.396 | 0.692 |
| Birth year: 2013 (N=4) | -0.147 | 0.111 | 1002 | -1.316 | 0.189 |
| Birth year: 2014 (N=2) | -0.242 | 0.193 | 1002 | -1.255 | 0.210 |
| Sample year: 2011 (N=178) | 0.042 | 0.026 | 1002 | 1.573 | 0.116 |
| Sample year: 2012 (N=238) | 0.063 | 0.033 | 1002 | 1.895 | 0.058 |
| Sample year: 2013 (N=269) | 0.087 | 0.045 | 1002 | 1.954 | 0.051 |
| Sample year: 2014 (N=246) | 0.064 | 0.06 | 1002 | 1.052 | 0.293 |
| sample interval in days | 0.000 | 0.000 | 1002 | -0.049 | 0.961 |
| Health event within 2 weeks of sampling | 0.018 | 0.024 | 1002 | 0.721 | 0.471 |

Table S2: Corresponding ANOVA output to Table S1.

|  | Sum Sq | Mean Sq | NumDF | DenDF | F value | p-value |
| --- | --- | --- | --- | --- | --- | --- |
| Age in years | 0.08 | 0.08 | 1 | 1002 | 2.57 | 0.110 |
| Feed group | 0.01 | 0.01 | 2 | 1002 | 0.25 | 0.780 |
| Genetic group | 0.02 | 0.02 | 1 | 1002 | 0.80 | 0.371 |
| Birth year | 0.13 | 0.02 | 6 | 1002 | 0.73 | 0.628 |
| Sample year | 0.31 | 0.08 | 4 | 1002 | 2.64 | 0.032 |
| sample interval in days | 7.21E-05 | 7.21E-05 | 1 | 1002 | 0.00 | 0.961 |
| Health event within 2 weeks of sampling | 0.01 | 0.02 | 1 | 1002 | 0.52 | 0.471 |

Table S3: Reduced model. Effect sizes and significance of fixed effects in linear mixed model of RLTL change, adjusted for qPCR plate and row with animal identity fitted as a random effect.

|  | Estimate | Std. Error | df | t value | p-value |
| --- | --- | --- | --- | --- | --- |
| (Intercept) | -0.142 | 0.028 | 1009 | -5.029 | <0.001 |
| Age in years | 0.026 | 0.006 | 1009 | 4.266 | <0.001 |
| Feed group: high forage (N=110) | -0.010 | 0.012 | 1009 | -0.867 | 0.386 |
| Feed group: not allocated (N= 80) | -0.015 | 0.015 | 1009 | -0.969 | 0.333 |
| Genetic group: select (N=135) | -0.013 | 0.011 | 1009 | -1.113 | 0.266 |
| Sample year: 2011 (N=178) | 0.040 | 0.023 | 1009 | 1.744 | 0.082 |
| Sample year: 2012 (N=238) | 0.061 | 0.022 | 1009 | 2.735 | 0.006 |
| Sample year: 2013 (N=269) | 0.081 | 0.024 | 1009 | 3.438 | 0.001 |
| Sample year: 2014 (N=246) | 0.050 | 0.027 | 1009 | 1.834 | 0.067 |
| sample interval in days | 0.000 | 0.000 | 1009 | 0.198 | 0.843 |

Table S4: Corresponding ANOVA output to Table S3.

|  | Sum Sq | Mean Sq | NumDF | DenDF | F value | p-value |
| --- | --- | --- | --- | --- | --- | --- |
| Age in years | 0.536 | 0.536 | 1 | 1009 | 18.200 | <0.001 |
| Feed group | 0.036 | 0.018 | 2 | 1009 | 0.619 | 0.539 |
| Genetic group | 0.037 | 0.037 | 1 | 1009 | 1.239 | 0.266 |
| Sample year | 0.452 | 0.113 | 4 | 1009 | 3.836 | 0.004 |
| Sample interval in days | 0.001 | 0.001 | 1 | 1009 | 0.039 | 0.843 |

|  | Estimate | Std. Error | df | t value | p-value |
| --- | --- | --- | --- | --- | --- |
| (Intercept) | -0.143 | 0.035 | 906 | -4.057 | <0.001 |
| Age in years | 0.021 | 0.007 | 906 | 3.078 | 0.002 |
| Feed group: high forage (N=108) | -0.006 | 0.013 | 906 | -0.429 | 0.668 |
| Feed group: not allocated (N=29) | 0.009 | 0.024 | 906 | 0.379 | 0.704 |
| Genetic group: select (N=117) | -0.011 | 0.012 | 906 | -0.926 | 0.355 |
| Sample year: 2011 (N=80) | 0.038 | 0.024 | 906 | 1.597 | 0.111 |
| Sample year: 2012 (N=75) | 0.06 | 0.023 | 906 | 2.6 | 0.009 |
| Sample year: 2013 (N=13) | 0.086 | 0.025 | 906 | 3.417 | 0.001 |
| Sample year: 2014 (N=4) | 0.064 | 0.03 | 906 | 2.131 | 0.033 |
| Sample interval in days | 0.000 | 0.000 | 906 | -0.461 | 0.645 |
| Milk productivity in kg /1000 | 0.003 | 0.003 | 906 | 0.963 | 0.336 |

Table S5: Reduced model including milk productivity. Linear mixed model of RLTL change, adjusted for qPCR plate and row with animal identity fitted as a random effect.

Table S6: Corresponding ANOVA output to Table S5.

|  | Sum Sq | Mean Sq | NumDF | DenDF | F value | p-value |
| --- | --- | --- | --- | --- | --- | --- |
| Age in years | 0.278 | 0.278 | 1 | 906 | 9.471 | 0.002 |
| Feed group | 0.018 | 0.009 | 2 | 906 | 0.299 | 0.742 |
| Genetic group | 0.025 | 0.025 | 1 | 906 | 0.857 | 0.355 |
| Sample year | 0.405 | 0.101 | 4 | 906 | 3.456 | 0.008 |
| sample interval in days | 0.006 | 0.006 | 1 | 906 | 0.212 | 0.645 |
| Milk productivity in kg/1000 | 0.0272 | 0.027 | 1 | 906 | 0.928 | 0.336 |

Table S7: Effect sizes and significance of fixed effects of age at second sampling from linear mixed-effects model of RLTL change, adjusted for qPCR plate and row with animal identity fitted as a random effect, Age at second sampling in years was tested as a factor to illustrate that average RLTL change across consecutive measurements was only significantly negative (indicating a tendency for attrition over time) when the first measurement was made close to birth and the follow up measurement at the age of around 1 year. For three animals, a second measurement was taken close to 0 years (birth) which explains why this age group appears in this table. The age of one year was set for this model as reference (representing the model intercept). All other ages are treated in relation to a change that has 1 year as second age measure. In comparison all other age groups show less negative (=in comparison positive) change. This does not mean that lengthening is observed.

| Age at second sampling | Estimate | Std. Error | df | t-value | p-value |
| --- | --- | --- | --- | --- | --- |
| 1 years | -0.115 | 0.01 | 1013 | -11.512 | <0.001 |
| 0 years | 0.015 | 0.065 | 1013 | 0.225 | 0.822 |
| 2 years | 0.106 | 0.014 | 1013 | 7.474 | <0.001 |
| 3 years | 0.127 | 0.015 | 1013 | 8.444 | <0.001 |
| 4 years | 0.111 | 0.017 | 1013 | 6.44 | <0.001 |
| 5 years | 0.142 | 0.025 | 1013 | 5.703 | <0.001 |
| 6 years | 0.089 | 0.039 | 1013 | 2.267 | 0.024 |

Table S8: Association of maximum summer temperature with change in RLTL

|  | Estimate | Std. Error | df | t value | p-value |
| --- | --- | --- | --- | --- | --- |
| (Intercept) | 0.093 | 0.062 | 1012 | 1.484 | 0.138 |
| Age in years | 0.028 | 0.004 | 1012 | 6.444 | <0.001 |
| Feed group: high forage | -0.011 | 0.012 | 1012 | -0.911 | 0.362 |
| Feed group: not allocated | -0.012 | 0.015 | 1012 | -0.806 | 0.420 |
| Genetic group: select | -0.012 | 0.011 | 1012 | -1.068 | 0.286 |
| Sample interval in days | 0.000 | 0.000 | 1012 | 0.326 | 0.745 |
| Max temperature in summer quarter | -0.012 | 0.004 | 1012 | -3.279 | 0.001 |

Table S9: Corresponding ANOVA output to Table S8.

|  | Sum Sq | Mean Sq | NumDF | DenDF | F value | p-value |
| --- | --- | --- | --- | --- | --- | --- |
| Age in years | 1.226 | 1.226 | 1 | 1012 | 41.530 | <0.001 |
| Feed group | 0.0319 | 0.016 | 2 | 1012 | 0.540 | 0.583 |
| Genetic group | 0.034 | 0.034 | 1 | 1012 | 1.140 | 0.286 |
| Sample interval in days | 0.003 | 0.003 | 1 | 1012 | 0.106 | 0.745 |
| Max temperature in summer quarter | 0.317 | 0.317 | 1 | 1012 | 10.751 | 0.001 |

Table S10: Sample year becomes non-significant when maximum summer temperature is in the same model.

|  | Estimate | Std. Error | df | t value | p-value |
| --- | --- | --- | --- | --- | --- |
| (Intercept) | -11.045 | 13.79 | 1011 | -0.801 | 0.423 |
| Age in years | 0.025 | 0.006 | 1011 | 4.129 | <0.001 |
| Feed group: high forage | -0.011 | 0.012 | 1011 | -0.875 | 0.382 |
| Feed group: not allocated | -0.014 | 0.015 | 1011 | -0.881 | 0.378 |
| Genetic group: select | -0.013 | 0.011 | 1011 | -1.136 | 0.256 |
| Sample interval in days | 0.000 | 0.000 | 1011 | 0.200 | 0.842 |
| Sample year | 0.006 | 0.007 | 1011 | 0.808 | 0.419 |
| Max temperature in summer quarter | -0.01 | 0.004 | 1011 | -2.523 | 0.012 |

Table S11: Corresponding ANOVA output to Table S10.

|  | Sum Sq | Mean Sq | NumDF | DenDF | F value | p-value |
| --- | --- | --- | --- | --- | --- | --- |
| Age in years | 0.503 | 0.503 | 1 | 1011 | 17.045 | <0.001 |
| Feed group | 0.033 | 0.017 | 2 | 1011 | 0.562 | 0.570 |
| Genetic group | 0.038 | 0.038 | 1 | 1011 | 1.291 | 0.256 |
| Sample interval in days | 0.001 | 0.001 | 1 | 1011 | 0.040 | 0.842 |
| Sample year | 0.019 | 0.019 | 1 | 1011 | 0.652 | 0.419 |
| Max temperature in summer quarter | 0.188 | 0.188 | 1 | 1011 | 6.365 | 0.012 |

Table S12: Association of sun hours per day averaged across the summer quarter with change in RLTL.

|  | Estimate | Std. Error | df | t value | Pr(>\|t\|) |
| --- | --- | --- | --- | --- | --- |
| (Intercept) | 0.06 | 0.072 | 1012 | 0.827 | 0.408 |
| Age in years | 0.025 | 0.005 | 1012 | 4.868 | <0.001 |
| Feed group: high forage | -0.011 | 0.012 | 1012 | -0.922 | 0.357 |
| Feed group: not allocated | -0.016 | 0.015 | 1012 | -1.005 | 0.315 |
| Genetic group: select | -0.014 | 0.011 | 1012 | -1.21 | 0.227 |
| Sample interval in days | 0.000 | 0.000 | 1012 | -0.105 | 0.917 |
| Mean sun hours in summer quarter | -0.001 | 0.000 | 1012 | -2.309 | 0.021 |

Table S13: Corresponding ANOVA output to Table S12.

|  | Sum Sq | Mean Sq | NumDF | DenDF | F value | p-value |
| --- | --- | --- | --- | --- | --- | --- |
| Age in years | 0.703 | 0.703 | 1 | 1012 | 23.698 | <0.001 |
| Feed group | 0.040 | 0.020 | 2 | 1012 | 0.6791 | 0.507 |
| Genetic group | 0.043 | 0.043 | 1 | 1012 | 1.463 | 0.227 |
| Sample interval in days | 0.000 | 0.000 | 1 | 1012 | 0.011 | 0.9167 |
| Mean sun hours in summer quarter | 0.158 | 0.158 | 1 | 1012 | 5.331 | 0.021 |

Table S14: Association of summer rain with change in RLTL.

|  | Estimate | Std. Error | df | t value | Pr(>\|t\|) |
| --- | --- | --- | --- | --- | --- |
| (Intercept) | -0.134 | 0.029 | 1012 | -4.612 | <0.001 |
| Age in years | 0.029 | 0.004 | 1012 | 6.446 | <0.001 |
| Feed group: high forage | -0.011 | 0.012 | 1012 | -0.95 | 0.342 |
| Feed group: not allocated | -0.012 | 0.015 | 1012 | -0.783 | 0.434 |
| Genetic group: select | -0.012 | 0.011 | 1012 | -1.093 | 0.275 |
| Sample interval in days | 0.000 | 0.000 | 1012 | 0.208 | 0.835 |
| Mean rain (mm) in summer quarter | 0.000 | 0.000 | 1012 | 2.051 | 0.041 |

Table S15: Corresponding ANOVA output to Table S14.

|  | Sum Sq | Mean Sq | NumDF | DenDF | F value | p-value |
| --- | --- | --- | --- | --- | --- | --- |
| Age in years | 1.234 | 1.234 | 1 | 1012 | 41.554 | <0.001 |
| Feed group | 0.033 | 0.017 | 2 | 1012 | 0.556 | 0.574 |
| Genetic group | 0.035 | 0.035 | 1 | 1012 | 1.195 | 0.275 |
| Sample interval in days | 0.001 | 0.001 | 1 | 1012 | 0.043 | 0.835 |
| Mean rain (mm) in summer quarter | 0.125 | 0.125 | 1 | 1012 | 4.207 | 0.041 |

Table S16: Association of maximum winter temperature with change in RLTL.

|  | Estimate | Std. Error | df | t value | p-value |
| --- | --- | --- | --- | --- | --- |
| (Intercept) | 0.069 | 0.068 | 1012 | 1.014 | 0.311 |
| Age in years | 0.032 | 0.004 | 1012 | 7.416 | <0.001 |
| Feed group: high forage | -0.012 | 0.012 | 1012 | -0.984 | 0.325 |
| Feed group: not allocated | -0.014 | 0.015 | 1012 | -0.931 | 0.352 |
| Genetic group: select | -0.012 | 0.011 | 1012 | -1.063 | 0.288 |
| Sample interval in days | 0.000 | 0.000 | 1012 | 0.11 | 0.912 |
| Max temperature in winter quarter | -0.014 | 0.005 | 1012 | -2.612 | 0.009 |

Table S17: Corresponding ANOVA output to Table S16.

|  | Sum Sq | Mean Sq | NumDF | DenDF | F value | Pr(>F) |
| --- | --- | --- | --- | --- | --- | --- |
| Age in years | 1.629 | 1.629 | 1 | 1012 | 54.996 | <0.001 |
| Feed group | 0.0395 | 0.020 | 2 | 1012 | 0.667 | 0.513 |
| Feed group | 0.034 | 0.034 | 1 | 1012 | 1.131 | 0.288 |
| Sample interval in days | 0.000 | 0.000 | 1 | 1012 | 0.012 | 0.912 |
| Max temperature in winter quarter | 0.202 | 0.202 | 1 | 1012 | 6.821 | 0.009 |

Table S18: Association of maximum summer and winter temperature with change in RLTL.

|  | Estimate | Std. Error | df | t value | p-value |
| --- | --- | --- | --- | --- | --- |
| (Intercept) | 0.140 | 0.074 | 1011 | 1.882 | 0.060 |
| Age in years | 0.029 | 0.004 | 1011 | 6.548 | <0.001 |
| Feed group: high forage | -0.011 | 0.012 | 1011 | -0.919 | 0.359 |
| Feed group: not allocated | -0.013 | 0.015 | 1011 | -0.856 | 0.392 |
| Genetic group: select | -0.012 | 0.011 | 1011 | -1.059 | 0.290 |
| Sample interval in days | 0.000 | 0.000 | 1011 | 0.298 | 0.766 |
| Max temperature in winter quarter | -0.007 | 0.006 | 1011 | -1.171 | 0.242 |
| Max temperature in summer quarter | -0.009 | 0.004 | 1011 | -2.297 | 0.022 |

Table S19: Corresponding ANOVA output to Table S18.

|  | Sum Sq | Mean Sq | NumDF | DenDF | F value | p-value |
| --- | --- | --- | --- | --- | --- | --- |
| Age in years | 1.265 | 1.265 | 1 | 1011 | 42.878 | <0.001 |
| Feed group | 0.034 | 0.017 | 2 | 1011 | 0.573 | 0.564 |
| Genetic group | 0.033 | 0.033 | 1 | 1011 | 1.120 | 0.290 |
| Sample interval in days | 0.003 | 0.003 | 1 | 1011 | 0.089 | 0.766 |
| Max temperature in winter quarter | 0.040 | 0.040 | 1 | 1011 | 1.370 | 0.242 |
| Max temperature in summer quarter | 0.156 | 0.156 | 1 | 1011 | 5.276 | 0.022 |

Table S20: Association of disease events (lameness and mastitis separately and together) on RLTL change tested in a linear model.

| lifetime RLTL change measure | Estimate | SE | t-value | p-value | Disease |
| --- | --- | --- | --- | --- | --- |
| mean RLTL | 0.000 | 0.005 | 0.08 | 0.937 | Lameness |
| mean RLTL change | 0.005 | 0.003 | 1.66 | 0.099 |  |
| mean absolute RLTL change | -0.004 | 0.003 | -1.31 | 0.192 |  |
| mean RLTL | -0.006 | 0.008 | -0.78 | 0.436 | Mastitis |
| mean RLTL change | 0.000 | 0.005 | 0.05 | 0.963 |  |
| mean absolute RLTL change | -0.007 | 0.005 | -1.32 | 0.190 |  |
| mean RLTL | -0.001 | 0.004 | -0.325 | 0.746 | Lameness + Mastitis |
| mean RLTL change | 0.004 | 0.003 | 1.382 | 0.169 |  |
| mean absolute RLTL change | -0.004 | 0.003 | -1.74 | 0.08 |  |

Table S21: Results of cox proportional hazards models testing association of relative leukocyte telomere length (RLTL) change measurements (on continuous scales) with productive lifespan. SE= standard error, CI = confidence interval.

| RLTL measure | Coefficient (SE) | Hazard ratio | 95% CI  (hazard ratio) | exp(-Coefficient) | z | p-value |
| --- | --- | --- | --- | --- | --- | --- |
| 1. **RLTL change within the first year of life (N=291)** | | | | | | |
| RLTL change | -1.141 (0.391) | 0.320 | 0.148 -0.688 | 3.129 | -2.914 | 0.004 |
| 1. **Each RLTL change measurements is tested in separate models (N=305)** | | | | | | |
| Mean RLTL | 0.341 (0.591) | 1.406 | 0.442-4.473 | 0.711 | 0.577 | 0.564 |
| Mean RLTL change | -5.209 (0.845) | 0.005 | 0.001-0.029 | 183 | -6.166 | <0.001 |
| Mean absolute RLTL change | 2.939 (0.970) | 18.8982 | 2.824-126.5 | 0.053 | 3.031 | 0.002 |
| 1. **All RLTL change measurements are tested in the same model (N=305)** | | | | | | |
| Mean RLTL | 0.053 (0.606) | 1.054 | 0.321-3.456 | 0.9488 | 0.087 | 0.931 |
| Mean RLTL change | -4.758 (1.018) | 0.009 | 0.001-0.063 | 116.722 | -4.676 | <0.001 |
| Mean absolute RLTL change | 0.776 (1.076) | 2.173 | 0.264-17.910 | 0.460 | 0.721 | 0.471 |

Table S22: Results of cox proportional hazard models of productive lifespan that correspond to the data shown in Figure 3 and Figure 4. RLTL change measures were transformed to a discrete scale with 3 groups as shown in Figures 3 and 4 to allow visualisation.

| Model | Coefficientt (SE) | Hazard ratio | 95% CI  (hazard ratio) | Exp(-coef) | z | p-value |
| --- | --- | --- | --- | --- | --- | --- |
| RLTL change within the first year of life (N=291) | -0.226 (0.082) | 0.798 | 0.6793  -  0.9369 | 1.253 | -2.755 | 0.006 |
| Mean RLTL (N = 305) | 0.014 (0.079) | 1.014 | 0.869  - 1.184 | 0.986 | 0.179 | 0.858 |
| Mean absolute RLTL change  (N = 305) | 0.179 (0.082) | 1.196 | 1.019  - 1.405 | 0.836 | 2.19 | 0.029 |
| Mean RLTL change  (N = 305) | -0.257 (0.087) | 0.773 | 0.652  - 0.916 | 1.294 | -2.974 | 0.003 |

Table S23: Results of cox proportional hazards models testing association of relative leukocyte telomere length (RLTL) change measurements (on continuous scales) with productive lifespan, including measures of average lifetime milk production. Average lifetime milk production was tested in the same models to account for the fact that cows may be culled because of poor productivity. SE= standard error, CI = confidence interval.

| Factor | Coefficient (SE) | Hazard ratio | 95% CI  (hazard ratio) | exp(-Coefficient) | z | p-value |
| --- | --- | --- | --- | --- | --- | --- |
| 1. **Mean RLTL, N=253** | | | | | | |
| Mean RLTL | 0.232 (0.671) | 1.260 | 0.339- 4.693 | 0.793 | 0.345 | 0.730 |
| Average lifetime milk production in kg / 1000 | -9.933 *10^-5^ (2.822 *10^-5^) | 1.000 | 0.9998-1.000 | 1.0001 | -3.520 | <0.001 |
| 1. **Mean RLTL change, N=253** | | | | | | |
| Mean RLTL change | -5.035 (1.315) | 0.007 | 0.001-0.086 | 153.7 | -3.829 | <0.001 |
| Average lifetime milk production in kg / 1000 | -9.057 *10^-5^  (2.819 *10^-5^) | 1.000 | 0.0005-0.086 | 1.000 | -3.212 | 0.001 |
| 1. **Mean absolute RLTL change, N=253** | | | | | | |
| Mean absolute RLTL change | 2.411 (1.146) | 11.15 | 1.1793- 105.4 | 0.090 | 2.104 | 0.035 |
| Average lifetime milk production in kg / 1000 | -1.018 *10^-4^ (2.818 *10^-5^) | 1.000 | 1.000-1.000 | 1.000 | -3.611 | <0.001 |
| 1. **All telomere change measures tested in the same model, N=253** | | | | | | |
| Mean RLTL | -0.070 | 0.933 | 0.244-3.564 | 1.072 | -0.102 | 0.919 |
| Mean RLTL change | -4.525 (1.387) | 0.011 | 0.001-0.164 | 92.303 | -3.262 | 0.001 |
| Mean absolute RLTL change | 1.237 (1.224) | 3.447 | 0.313-37.945 | 0.290 | 1.011 | 0.312 |
| Average lifetime milk production in kg / 1000 | -9.162 *10^-5^  (2.824 *10^-5^) | 1.000 | 1.000-1.000 | 1.000 | -3.244 | 0.001 |

Table S24: Results of cox proportional hazards models testing association of relative leukocyte telomere length (RLTL) change measurements (on continuous scales) with productive lifespan, with first RLTL measurements (obtained close to birth) removed from analyses. N=253; SE= standard error, CI = confidence interval.

| Factor | Coefficient (SE) | Hazard ratio | 95% CI  (hazard ratio) | exp(-Coefficient) | z | p-value |
| --- | --- | --- | --- | --- | --- | --- |
| 1. **Mean RLTL, N=253** | | | | | | |
| Mean RLTL | -0.485 (0.649) | 0.616 | 0.173-2.199 | 1.624 | -0.746 | 0.455 |
| Average lifetime milk production in kg / 1000 | -9.853 * 10^-5^  (2.812 * 10^-5^) | 1.000 | 1.000-1.000 | 1.000 | -3.504 | <0.001 |
| 1. **Mean RLTL change, N=253** | | | | | | |
| Mean RLTL change | -5.056 (1.315) | 0.006 | 0.0005-0.084 | 153.7 | -3.829 | <0.001 |
| Average lifetime milk production  in kg / 1000 | -9.057* 10^-5^  (2.818* 10^-5^) | 1.000 | 1.000-1.000 | 1.000 | -3.228 | 0.001 |
| 1. **Mean absolute RLTL change, N=253** | | | | | | |
| Mean absolute RLTL change | 2.403 (1.147) | 11.05 | 1.168-104.6 | 0.090 | 2.095 | 0.036 |
| Average lifetime milk production  in kg / 1000 | -1.018* 10^-4^ (2.818* 10^-5^) | 1.000 | 1.000-1.000 | 1.000 | -3.610 | <0.001 |
| **4) Mean RLTL change measures tested in the same model, N=253** | | | | | | |
| Mean RLTL | -0.209 (0.670) | 0.812 | 0.218-3.017 | 1.232 | -0.311 | 0.756 |
| Mean RLTL change | -4.444 (1.429) | 0.012 | 0.001-0.194 | 85.107 | -3.109 | 0.002 |
| Mean absolute RLTL change | 1.281 (1.217) | 3.600 | 0.331-39.130 | 0.278 | 1.052 | 0.293 |
| Average lifetime milk production  in kg / 1000 | -9.208* 10^-5^ (2.820* 10^-5^) | 1.000 | 1.000-1.000 | 1.000 | -3.265 | 0.001 |

Table S25: Results of Cox proportional hazards models testing for independent associations of mean relative leukocyte telomere length (RLTL) change measurements and RLTL measured at 1 year of age on productive lifespan. Mean RLTL change remains highly statistically significant whereas RLTL at the age of 1 year becomes non-significant; SE= standard error, CI = confidence interval.

| Factor | Coefficient (SE) | Hazard ratio | 95% CI  (hazard ratio) | exp(-Coefficient) | z | p-value |
| --- | --- | --- | --- | --- | --- | --- |
| Mean RLTL  Change | -5.030 (1.379) | 0.007 | 0.0004-0.098 | 152.972 | -3.648 | <0.001 |
| RLTL at the age of 1 year | -0.655 (0.526) | 0.519 | 0.185-1.456 | 1.926 | -1.245 | 0.213 |
| Average lifetime milk production  in kg / 1000 | -7.896 *10^-5^  (2.928 *10^-5^) | 0.999 | 0.999-0.999 | 1.000 | -2.696 | 0.007 |

Table S26: Results of Cox proportional hazard model for lifetime RLTL dynamics measurements (mean RLTL, mean RLTL change and mean absolute RLTL change) restricted to animals with a known, disease-related reasons for culling. Animals that were culled due to accidents, due to herd management decisions and animals for which the reason for culling was unknown were excluded.

| Factor | Coefficient (SE) | Hazard ratio | 95% CI  (hazard ratio) | exp(-Coefficient) | z | p-value |
| --- | --- | --- | --- | --- | --- | --- |
| RLTL change within the first year of life, N=230 | -0.271 (0.096) | 0.762 | 0.631-0.920 | 1.312 | -2.822 | 0.005 |
| Mean RLTL, N=238 | 0.0129 (0.091) | 1.013 | 0.847- 1.212 | 0.987 | 0.141 | 0.888 |
| Mean RLTL change, N=238 | -0.290 (0.103) | 0.748 | 0.612- 0.915 | 1.337 | -2.825 | 0.005 |
| Mean absolute RLTL change, N=238 | 0.185 (0.097) | 1.203 | 0.995- 1.455 | 0.831 | 1.911 | 0.056 |
