# Supplementary material for: Telomere attrition rates are associated with weather conditions and predict productive lifespan in dairy cattle
Source: Sci Rep. 2021 Mar 10;11:5589. doi: 10.1038/s41598-021-84984-2 (PMC7970942; doi:10.1038/s41598-021-84984-2)
Supplement: Supplementary file 2 — Supplementary Information 2. [file 41598_2021_84984_MOESM2_ESM.docx]

**Supplementary File 2**

## Detailed Materials and Methods

# Telomere attrition rates are associated with weather conditions and predict productive lifespan in dairy cattle

**Authors & Affiliations:**

Luise A. Seeker^1,2*^, Sarah L. Underwood^3^, Rachael V. Wilbourn^3^, Jennifer Dorrens ^3^, Hannah Froy^3,4^, Rebecca Holland^3^, Joanna J. Ilska^1, 5^, Androniki Psifidi^5,6^, Ainsley Bagnall^7^, Bruce Whitelaw^5^, Mike Coffey^1^, Georgios Banos^1, 5^ & Daniel H. Nussey^3^

^1^ Animal & Veterinary Sciences, SRUC, Roslin Institute Building, Easter Bush, Midlothian, UK

^2^ MRC Centre for Regenerative Medicine, University of Edinburgh, Edinburgh, UK

^3^ Institute of Evolutionary Biology, School of Biological Sciences, University of Edinburgh, UK

^4^ Centre for Biodiversity Dynamics, NTNU Norwegian University of Science and Technology, Trondheim, Norway

^5^ The Roslin Institute and Royal (Dick) School of Veterinary Studies, University of Edinburgh, Easter Bush, Midlothian, UK

^6^ Royal Veterinary College, University of London, Hatfield, UK

^7^ SRUC Crichton Royal Farm, Glencaple Road, Dumfries, UK

### Animal population and data collection

At the Crichton Royal Farm, 200 milking cows plus their calves and replacement heifers are kept at any time. One half of the milking cows belong to a genetic line that has been selected for high milk protein and fat yield (S), while the other half is deliberately maintained on a UK average productivity level (C). Selection for these two genetic lines started in the 1970s. Animals of the C and S line do not significantly differ in their frame, weight or body condition score (p>0.05) as determined using t-tests for the animals in the present study. Each new-born calf is weighed and ear-marked and kept in an individual housing for the first few days of its life. Then C and S calves are transferred to outsides pens with a shelter, where groups of calves live together. All heifers are managed in the same way until their first calving when they are randomly allocated to a high forage (HF) or low forage (LF) diet. The LF diet is based on human food by-products and consists of a concentrate blend. The HF diet on the other hand is based on feed that is grown at the Crichton Royal Farm. While cows on a HF diet are turned out over the summer months for grazing and are only housed over the winter months, cows on a LF diet are housed continuously over the year without a grazing period. The food and water consumption of all calves and cows is monitored. All cows are milked three times daily and milk yield, milk composition and the milk somatic cell count as an indicator for (subclinical) mastitis are recorded. In the present study, these measurements were used to calculate an average milk production in kg per cow including all started lactations. The average of these measurements per cow across their lifetime was calculated and is referred to below as “average lifetime milk productivity” (Figure S2). Every day cows leave the milking parlour over a pressure plate which detects signs of lameness. Behaviour and health events are documented after visual detection by farm workers (Figure S7). At the end of the animal’s life its productive lifespan and a reason for culling is recorded. Productive lifetime is the time from birth to culling in days and is a proxy for the health span of the animal, because all animals that remain healthy enough to generate profit for the farmer remain in the herd. The most frequent reasons for culling were reproductive problems, mastitis and, lameness (Figure S8). Along with a plethora of data that is recorded for each animal, routine blood sampling takes place initially shortly after birth and then annually in spring by venepuncture (V. jugularis for calves, V. caudalis for cows). Because of this sampling routine and because calves are being born all year round, age at sampling and sampling intervals vary for adult animals (Figure S2 & Figure S4). Blood tubes contained EDTA as anti-coagulant and were frozen on the farm and stored at -30 °C. They were transported in transportable freezers or on dry ice to the laboratory and underwent no freeze-thaw cycle before DNA was extracted for telomere length measurement.

### DNA extraction and qPCR

DNA from whole blood samples was extracted with the DNeasy Blood and Tissue spin column kit (QIAGEN). All DNA samples (100 %) had to have a minimum yield, purity and integrity to pass our internal quality control. Yield and purity were measured on a NanoDrop ND-1000 spectrophotometer (Thermo Scientific) and the DNA integrity was evaluated on integrity gels following Seeker et al. (2016)^1^. In total, 1,328 samples of 308 animals with a minimal concentration of 20 ng/μl and ratios of 260/280 > 1.7 and 260/230 >1.8 that also had a DNA integrity score of 1 or 2^1^ were included on qPCR plates for RLTL measurements. We measured telomeric DNA in relation to the reference gene beta-2-microglobulin (B2M) that is constant in copy number^1^ and has been used before in telomere studies on ruminant species such as Soay sheep, Roe deer and dairy cattle^1–5^. Both reactions were performed on the same qPCR plate but in different wells (monoplex qPCR). An identical sample was included as a calibrator (or “golden sample”) twice on all 25 qPCR plates: one time in the middle of the plate and another time at its periphery. The measurements for the calibrator sample were used in the calculation of RLTL of individual samples to correct for part of the random measurement error that was associated with the qPCR plate. The two locations of the calibrator were used to test for a qPCR plate edge effect. All other samples were randomly allocated to qPCR plates and wells. Also, a negative control (DNAse and RNase free water) and a serial dilution of the calibrator DNA were added to each qPCR plate for visual qPCR quality control. A liquid handling robot (Freedom Evo by TECAN) was used to load samples, the calibrator, the negative control and the serial dilution in triplicates onto 384 well qPCR plates. For the amplification of telomeres tel 1b (5’-CGG TTT GTT TGG GTT TGG GTT TGG GTT TGG GTT TGG GTT-3’) and tel 2b (5’-GGC TTG CCT TAC CCT TAC CCT TAC CCT TAC CCT TAC CCT-3’) primers were used^6^. B2M primers were obtained from (Primerdesign, accession code NM_001009284, sequence is proprietary).

The following qPCR protocol was used on a LightCycler 480 (Roche): 15 min at 95 °C for enzyme activation followed by 50 cycles of 15 s at 95 °C (denaturation), 30 s at 58 °C (primer annealing) and 30 s at 72 °C (signal acquisition). The melting curve was acquired as follows: 1 min at 95 °C, followed by 30 s at 58 °C and a continuous increase of 0.11 °C/s to 95 °C with continuous signal acquisition.

The software LinReg PCR^7^ was used for fluorescence baseline correction of raw RLTL measurements and for the calculation of reaction specific qPCR efficiencies for each plate qPCR efficiencies ranged from 94.3% to 94.85% for the B2M reaction and from 91.5–95.85% for the telomere reaction. The following formula was used for RLTL calculation^8^:

$RLTL=\frac{E_{TEL}^{{Cq}_{TEL(Calibrator)}-{Cq}_{TEL(Sample)}}}{E_{B2M}^{{Cq}_{B2M(Calibrator)}-{Cq}_{B2M(Sample)}}}$ (1)

The Cq value describes the number of cycles of a qPCR that is required for an amplification curve to cross a set fluorescence threshold. The Cq values of the calibrator sample were Cq_TEL(Calibrator)_ and Cq_B2M(Calibrator)_ for the telomere and the B2M reaction respectively. Cq values of the individual samples were Cq_TEL(Sample)_ and Cq_B2M(Sample)_.

### Calculation of sample repeatability

The first experimental qPCR plate was run four times on two subsequent days and a linear mixed model that accounted for qPCR plate as fixed effect and included sample ID as random effect was used to calculate the sample repeatability as the variance due to the sample divided by the total variance. The sample repeatability was 0.8 which can be considered satisfactory for measuring interpretable results^9^. Pairwise correlations of all four plates ranged between 0.75 and 0.89.

## References

1. Seeker, L. A. *et al.* Method specific calibration corrects for DNA extraction method effects on relative telomere length measurements by quantitative PCR. *PLoS One* **11**, 1–15 (2016).

2. Froy, H. *et al.* No evidence for parental age effects on offspring leukocyte telomere length in free-living Soay sheep. *Sci. Rep.* **7**, 1–9 (2017).

3. Watson, R. L. *et al.* Sex differences in leucocyte telomere length in a free-living mammal. *Mol. Ecol.* **26**, 3230–3240 (2017).

4. Fairlie, J. *et al.* Lifelong leukocyte telomere dynamics and survival in a free-living mammal. *Aging Cell* 140–148 (2015) doi:10.1111/acel.12417.

5. Wilbourn, R. V. *et al.* Age-dependent associations between telomere length and environmental conditions in roe deer. *Biol. Lett.* **13**, 20170434 (2017).

6. Epel, E. S. *et al.* Accelerated telomere shortening in response to life stress. *Proc. Natl. Acad. Sci.* **101**, 17312–17315 (2004).

7. Ruijter, J. M. *et al.* Amplification efficiency: linking baseline and bias in the analysis of quantitative PCR data. *Nucleic Acids Res.* **37**, e45 (2009).

8. Pfaffl, M. W. A new mathematical model for relative quantification in real-time RT–PCR. *Nucleic Acids Res.* **29**, e45 (2001).

9. Nettle, D., Seeker, L., Nussey, D., Froy, H. & Bateson, M. Consequences of measurement error in qPCR telomere data: A simulation study. *bioRxiv* (2018) doi:10.1101/491944.
