# Supplementary material for: Telomere attrition rates are associated with weather conditions and predict productive lifespan in dairy cattle
Source: Sci Rep. 2021 Mar 10;11:5589. doi: 10.1038/s41598-021-84984-2 (PMC7970942; doi:10.1038/s41598-021-84984-2)
Supplement: Supplementary file 3 — Supplementary Information 3. [file 41598_2021_84984_MOESM3_ESM.dotx]

# Supplementary File 3: ARRIVE Guidelines

# Telomere attrition rates are associated with weather conditions and predict productive lifespan in dairy cattle

**Short running title:** Telomere length attrition predicts lifespan

**Authors & Affiliations:**

Luise A. Seeker^1,2*^, Sarah L. Underwood^3^, Rachael V. Wilbourn^3^, Jennifer Dorrens, Hannah Froy^3,4^, Rebecca Holland^3^, Joanna J. Ilska^1, 5^, Androniki Psifidi^5,6^, Ainsley Bagnall^7^, Bruce Whitelaw^5^, Mike Coffey^1^, Georgios Banos^1, 5^ & Daniel H. Nussey^3^

^1^ Animal & Veterinary Sciences, SRUC, Roslin Institute Building, Easter Bush, Midlothian, UK

^2^ MRC Centre for Regenerative Medicine, University of Edinburgh, Edinburgh, UK

^3^ Institute of Evolutionary Biology, School of Biological Sciences, University of Edinburgh, UK

^4^ Centre for Biodiversity Dynamics, NTNU Norwegian University of Science and Technology, Trondheim, Norway

^5^ The Roslin Institute and Royal (Dick) School of Veterinary Studies, University of Edinburgh, Easter Bush, Midlothian, UK

^6^ Royal Veterinary College, University of London, Hatfield, UK

^7^ SRUC Crichton Royal Farm, Glencaple Road, Dumfries, UK

***Corresponding author:**

Luise A. Seeker

MRC Centre for Regenerative Medicine*,* The University of Edinburgh*,* Edinburgh BioQuarter, 5 Little France Drive, Edinburgh EH16 4UU

[Luise.seeker@ed.ac.uk](mailto:Luise.seeker@ed.ac.uk)

07591133397

It is good practice for animal experiments to follow ARRIVE guidelines^1^ to allow readers to interpret results. Our study differs from experimental studies using lab animals as there were no procedures conducted, and except for the feeding groups, animals were not allocated to treatment groups. Our study was based on the selection of archived blood samples based on certain inclusion criteria and observed the health and survival of included animals. We aimed to provide ARRIVE guideline information throughout the manuscript, but additionally offer essential information in the table below:

| Arrive Essentials | | |
| --- | --- | --- |
| Study design | 1 | 1. From an archive of collected bovine whole blood samples, 1336 longitudinal samples of 308 female animals were selected. Animals were all Holstein Friesian cattle and belonged to two different genetic groups (one generated by selective breeding for high milk productivity (S), the other bred as a control group to reflect UK average productivity (C)). Animals of both groups were randomly allocated to a feeding group, one higher in energy (LF), the other higher in fibre (HF). Disease events and the time from birth to culling alongside a reason for culling were observed. 2. The experimental unit was depending on the analysis either the sample or the animal. |
| Sample size | 2 | 1. Genetic select (S): 172 animals,  Genetic control (C): 133 animals,  Feed group (HF): 110 Feed group (LF): 118 77 animals not allocated to feed group (typically done after first calving) 2. Sample size was decided by including all samples that fulfilled inclusion criteria. |
| Inclusion and exclusion criteria | 3 | 1. From an archive of more than 5000 whole blood samples those of female animals with at least one early-life sample (taken within 15 days after birth) and one follow up sample were selected. Samples were excluded, if they failed DNA quality control measurements (DNA yield, purity and integrity measures)^2^ or qPCR quality control^2^. Animals were excluded if they had no longitudinal samples remaining (< 2 samples) after sample quality control steps. 2. Eight samples failed DNA quality control steps and were therefore removed from downstream analysis. Three animals were removed for not having repeat samples. 3. The exact number of animals and samples for each analysis are reported throughout the manuscript and in all tables. |
| Randomisation | 4 | 1. Samples in our study were completely randomly allocated to DNA extraction batches and qPCR plates to avoid confounding of experimental groups with measurement batches. Randomisation was performed in Excel, by creating a column with random numbers (=rand()) and sorting the data frame by that column. Samples were then allocated a “process number” in order of the sorted data frame and this process number was used for labelling samples in the lab allowing to process them in a random order while being blinded regarding their origin. 2. Sample randomisation should prevent confounding measurement batches with experimental groups. Batch effects were further reduced by using a liquid handling robot for pipetting steps. |
| Blinding | 5 | Samples were processed in random order and only labelled with a process number while handled in the lab. During DNA extraction, qPCR and quality control steps it was not possible to connect a sample to an experimental group. |
| Outcome measures | 6 | Relative leukocyte telomere length, age at sampling and survival in days. |
| Statistical methods | 7 | Linear mixed models, Cox proportional hazard analysis, linear models |
| Experimental animals | 8 | Female Holstein Friesian Dairy cattle repeatedly sampled from birth to death/ culling. |
| Experimental procedures | 9 | No experimental procedures were performed, except for regular blood sampling shortly after birth, annually in spring and if possible shortly before death/ culling |
| Results | 10 | Results are reported throughout the manuscript. |

# References:

1. du Sert, N. P. *et al.* The arrive guidelines 2.0: Updated guidelines for reporting animal research. *PLoS Biol.* **18**, 1–12 (2020).

2. Seeker, L. A. *et al.* Bovine telomere dynamics and the association between telomere length and productive lifespan. *Sci. Rep.* (2018).
